# Cyclization in Linear Step-Growth Polymerizations

**DOI:** 10.1021/acs.macromol.5c00980

**Published:** 2025-09-11

**Authors:** Yinghao Li, Jing Lyu, Wenxin Wang

**Affiliations:** † Charles Institute of Dermatology, School of Medicine, 8797 University College Dublin, Dublin 4 D04V1W8, Ireland; ‡ Institute of Precision Medicine (AUST-IPM), Anhui University of Science and Technology, Huainan 232001, China

## Abstract

Intramolecular cyclization is a pervasive yet often ignored factor in step-growth polymerizations (SGPs), particularly under dilute conditions. While experimental studies have confirmed the significant impact of concentration on cyclization, the lack of deep theoretical understanding has limited the ability to guide reaction design and predict the polymer structure. In this work, we adopt a reverse-engineering strategy to extract cyclization-related equations from experimental data using a classical A2 + B2 step-growth polymerization system. By combining analytical derivations with symbolic regression, a machine learning technique for generating closed-form expressions, we obtain explicit formulas for cyclization probability, degree of cyclization, degree of linear polymerization, and molecular weights as functions of monomer conversion and reaction concentration. These expressions capture the dynamic nature of cyclization and demonstrate excellent agreement with experimental results across a broad concentration range. Our work provides a new quantitative framework to incorporate cyclization into SGP theory and offers practical tools for predicting molecular structures and properties under real-world conditions.

## Graphical abstract

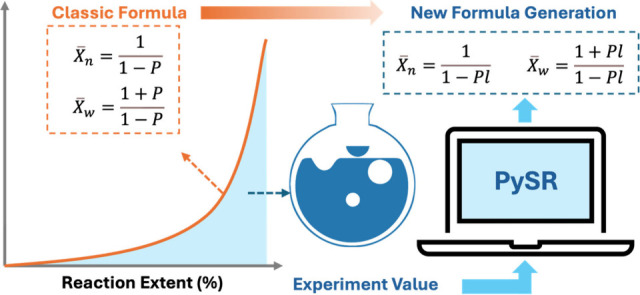

## Introduction

Step-growth polymerization is a fundamental polymerization method that has been widely applied in daily life and cutting-edge scientific research since the concept of polymers was first introduced. However, the study of its polymerization mechanism is far from complete. The foundational theory of step-growth polymerization, the Flory Principle, neglects the effects of intramolecular cyclization.^1,2^ In reality, cyclization reactions are almost unavoidable during step-growth polymerizations, especially under dilute reaction conditions.^3–5^ These reactions significantly influence the final structure and properties of polymers, especially under dilute conditions. For example, during the synthesis of high molecular weight linear polymers, cyclization can lead to a broader molecular weight distribution, thereby affecting the material’s mechanical and processing properties.^6,7^ Additionally, cyclization reactions are intentionally utilized in the synthesis of polymers with specific topologies, such as cyclic polymers, to endow materials with some unique physical and chemical properties.^8–10^ Therefore, in industrial production, precise control of polymerization conditions, such as monomer concentration, reaction time, and temperature, is crucial to control cyclization reactions. This not only helps in enhancing the yield and quality of the target polymer but also ensures that the material exhibits the expected performance in practical applications.

Over the past few decades, substantial research has been dedicated to understanding cyclization.^11–13^ Experimentally, the impact of reaction concentration on cyclization has been studied extensively, confirming that cyclization increases as the reaction concentration decreases.^14–21^ Additionally, in viscous systems, theoretical study and computational simulations have shown that the probability of cyclization increases as the reaction progresses.^22,23^ Although it is now widely accepted that dilution increases the probability of cyclization in step-growth polymerizations, it remains unfortunate that no deep understanding currently exists to relate experimental reaction concentration to the degree of cyclization or molecular weight. As a result, there is a lack of reliable theoretical guidance for reaction design. Furthermore, the probability of cyclization is inherently a dynamic physical parameter that varies continuously with the reaction environment, rather than a fixed value, adding further complexity to theoretical modeling.^23–25^ Obtaining elegant and practical expressions to describe cyclization probability under various reaction conditions has long been a major challenge in the field of step-growth polymerization.

In contrast to traditional approaches, which typically propose hypothetical models, derive theoretical equations, and then compare them with experimental data, this work takes a reverse-engineering strategy. Using the classical linear step-growth A2 + B2 polymerization as a representative example, we start from actual experimental results and extract cyclization-related equations under different reaction concentrations. This approach aims to uncover the evolving pattern of cyclization probability as a function of reaction conversion under varying experimental conditions. Moreover, due to the inherent difficulty in directly fitting experimental results across diverse conditions with traditional mathematical methods, this study employs symbolic regression, a technique from the field of machine learning. Our work provides concise and practical expressions for cyclization probability, degree of cyclization, degree of linear polymerization, and number-/weight-average molecular weights (*M̅*_*n*_, *M̅*_*w*_), as well as dispersity (*Đ*), all accounting for cyclization effects under different reaction concentrations.

## Results and Discussion

### Calculation of the Extent of Cyclization (Pc) and Linear Propagation (Pl)

According to the pioneering work by Flory and others, for an equimolar A2 + B2 step-growth polymerization system, the number-average degree of polymerization (*X̅*_*n*_) and weight-average degree of polymerization (*X̅*_*w*_) are classically described by the following equations:^1^

1 $${\mathrm{X̅}}_{n}=\frac{1}{1-P}$$

2 $${\mathrm{X̅}}_{w}=\frac{1+P}{1-P}$$

where P represents the extent of functional group conversion.

When intramolecular cyclization is taken into account, P can be expressed as

3 P=Pl+Pc

where Pl is the extent of linear propagation, denoting the functional group conversion consumed by linear growth reactions. Pc is the extent of cyclization, representing the functional group conversion consumed by cyclization reactions. Under these conditions, Equations 1 and 2 can be reformulated as

4 $${\mathrm{X̅}}_{n}=\frac{1}{1-Pl}$$

5 $${\mathrm{X̅}}_{w}=\frac{1+Pl}{1-Pl}$$

Based on the above equations, Pl can be calculated from experimentally measured *M̅*_*n*_ or *M̅*_*w*_:

6 $$Pl=1-\frac{{\mathrm{M̅}}_{0}}{{\mathrm{M̅}}_{w}}$$

7 $$Pl=1-\frac{2}{1+\frac{{\mathrm{M̅}}_{w}}{{\mathrm{M̅}}_{0}}}$$

where *M̅*_0_ is the average molecular weight of the A2 and B2 monomers.

Similarly, Pc can be obtained as

8 $$Pc=P-\left(1-\frac{{\mathrm{M̅}}_{0}}{{\mathrm{M̅}}_{w}}\right)$$

9 $$Pc=P-\left(1-\frac{2}{1+\frac{{\mathrm{M̅}}_{w}}{{\mathrm{M̅}}_{0}}}\right)$$

Therefore, by experimentally measuring *M̅*_*n*_ and *M̅*_*w*_ at different conversion levels P, one can derive the variation of Pc as a function of conversion under different reaction concentrations.

### Preparation and Synthesis of the A2 + B2 Model System

A classic A2 + B2 poly­(β-amino ester) system^26^ was selected for the experimental model, in which butanediol diacrylate (BDA) served as the A2 monomer and an equimolar amount of *N*,*N*′-dimethyl-1,6-hexanediamine (DHD) was used as the B2 monomer (Figure 1A). These monomers undergo polymerization via a simple and reliable Michael addition reaction.

**Figure 1:**
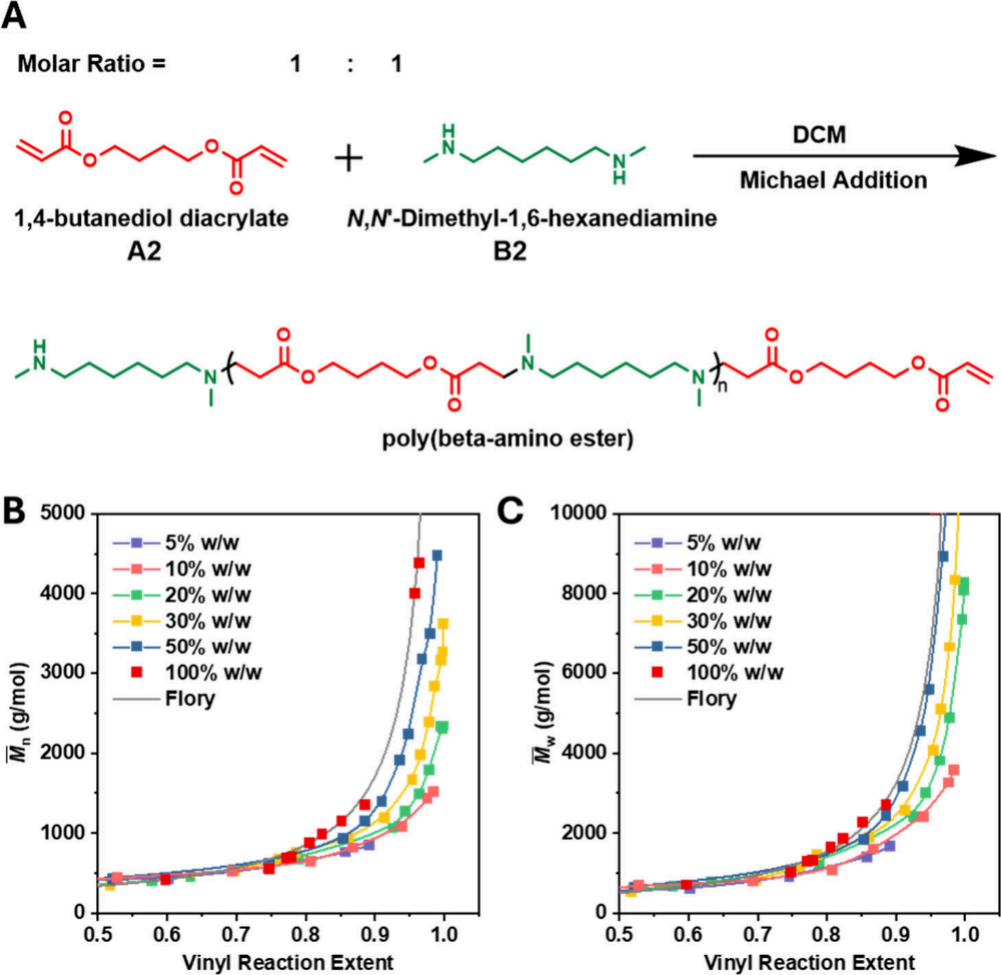
Experimental model and polymerization kinetics at different reaction concentrations. (A) Schematic representation of the chemical structures of the monomers and resulting polymer. (B) Kinetic curves of *M̅*_*n*_ and (C) *M̅*_*w*_ as a function of monomer conversion at various reaction concentrations. The ideal Flory reference curves were calculated by substituting the vinyl reaction extent into Equations 1 and 2 and then multiplying by *M̅*_0_.

Dichloromethane (DCM) was used as the solvent, and the reaction concentrations were set at 100%, 50%, 30%, 20%, 10%, and 5% weight-to-weight ratio (w/w). The molecular weight (*M̅*_*n*_ and *M̅*_*w*_) of the resulting polymers was monitored via gel permeation chromatography (GPC), with the detailed synthetic procedures provided in the Supporting Information (Figure S1). The overall conversion P, defined by the consumption of the acrylate double bonds on BDA, was monitored by ^1^H NMR spectroscopy (Figures S2–S7).

As shown in Figures 1B, 1C, a clear trend emerges: as the reaction concentration decreases, the extent of cyclization becomes increasingly pronounced. This is evidenced by the fact that, at comparable levels of monomer conversion, both *M̅*_*n*_ and *M̅*_*w*_ decrease with decreasing reaction concentration. Notably, the kinetic curve for the 5% w/w system shows a significant drop below the ideal Flory model reference, consistent with the widely recognized understanding that cyclization is more dominant under dilute conditions.

### Symbolic Regression Algorithm Program Construction

A program combining evolutionary search algorithms for formula optimization and identification was implemented using Python Symbolic Regression (PySR)^27^ to perform symbolic regression modeling (Scheme 1). Based on Equations 6–9, the values of *M̅*_*n*_ and *M̅*_*w*_ for each sample point were used to calculate the corresponding Pl and Pc, which served as the input data for the regression. In each training round, a random set of mathematical expressions was generated to form the initial population. These expressions were composed of various arithmetic operations and variables. Crossover and mutation operations were applied according to preset probabilities to explore the solution space. The generated expressions were then ranked based on a composite Score that evaluates both their Loss and complexity. High-ranking expressions were selected through survivor selection to enter the next generation. The evolutionary process continued until a stopping criterion was met, which was determined based on the Loss (mean squared error, MSE). The final formula was then output as the result of the regression.

**Scheme 1:**
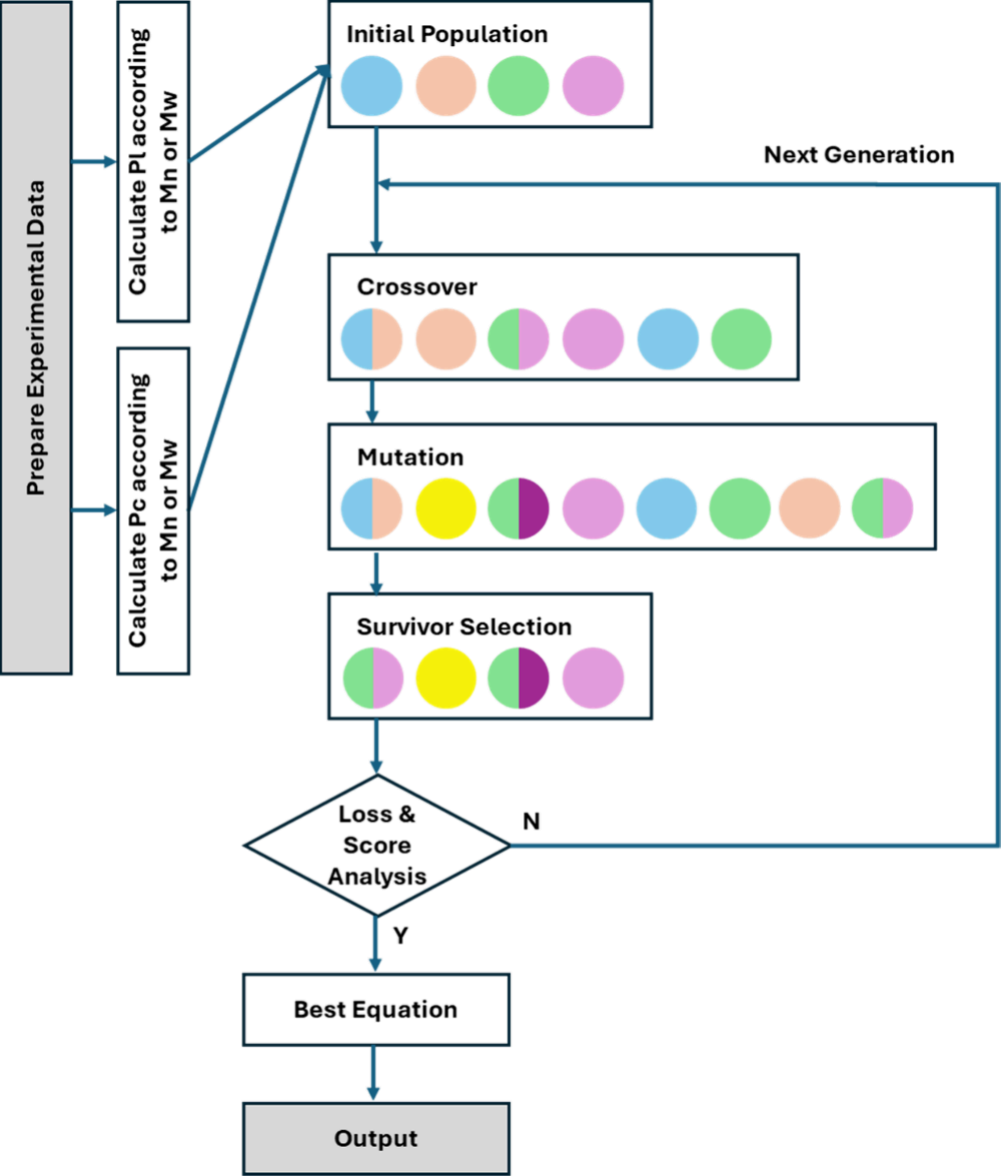
Symbolic Regression Algorithm

### Construction of Pl Formulas Using Different Configurations and Input Data

To derive an expression for Pl, representing the degree of linear growth consumption as a function of monomer conversion, we first used experimental data to calculate Pl at different conversion levels. To balance formula simplicity and accuracy, three different hyperparameter configurations were compared: (a) operators: + , -, *, /, maximum complexity = 20; (b) operators: + , -, *, /, maximum complexity = 100; (c) operators: + , -, *, /, exponentiation, square, power, square root, logarithm, cube, maximum complexity = 100. In the symbolic regression framework, formula complexity was defined as follows: using + , -, or a variable increased complexity by 1; * or/increased complexity by 2; power functions increased complexity by 3; and more complex operations such as exponentiation, logarithm, or cube increased complexity by 4. Additionally, two sets of input data were used: Pl values calculated from *M̅*_*n*_ and those from *M̅*_*w*_. The symbolic regression program was first performed using the Pl values derived from *M̅*_*n*_. The fitted formulas under configurations (a), (b), and (c) are presented in Tables S1, S2, and S3, respectively. As shown in Figures 2A–2C, when only basic arithmetic operators (+, -, *, /) were used (cases a and b), the Loss dropped rapidly. However, increasing the maximum allowed complexity did not lead to further significant reduction in Loss. Moreover, the best-performing formulas (highlighted in Tables S1, S2, ranked by Score) showed little improvement in prediction accuracy (Figure S8A vs S8B). Substituting these formulas into varying reaction concentrations and conversion levels revealed outliers and unstable predictions in the Pl curves (Figure S9A, S9B), indicating overfitting and poor generalizability.

**Figure 2:**
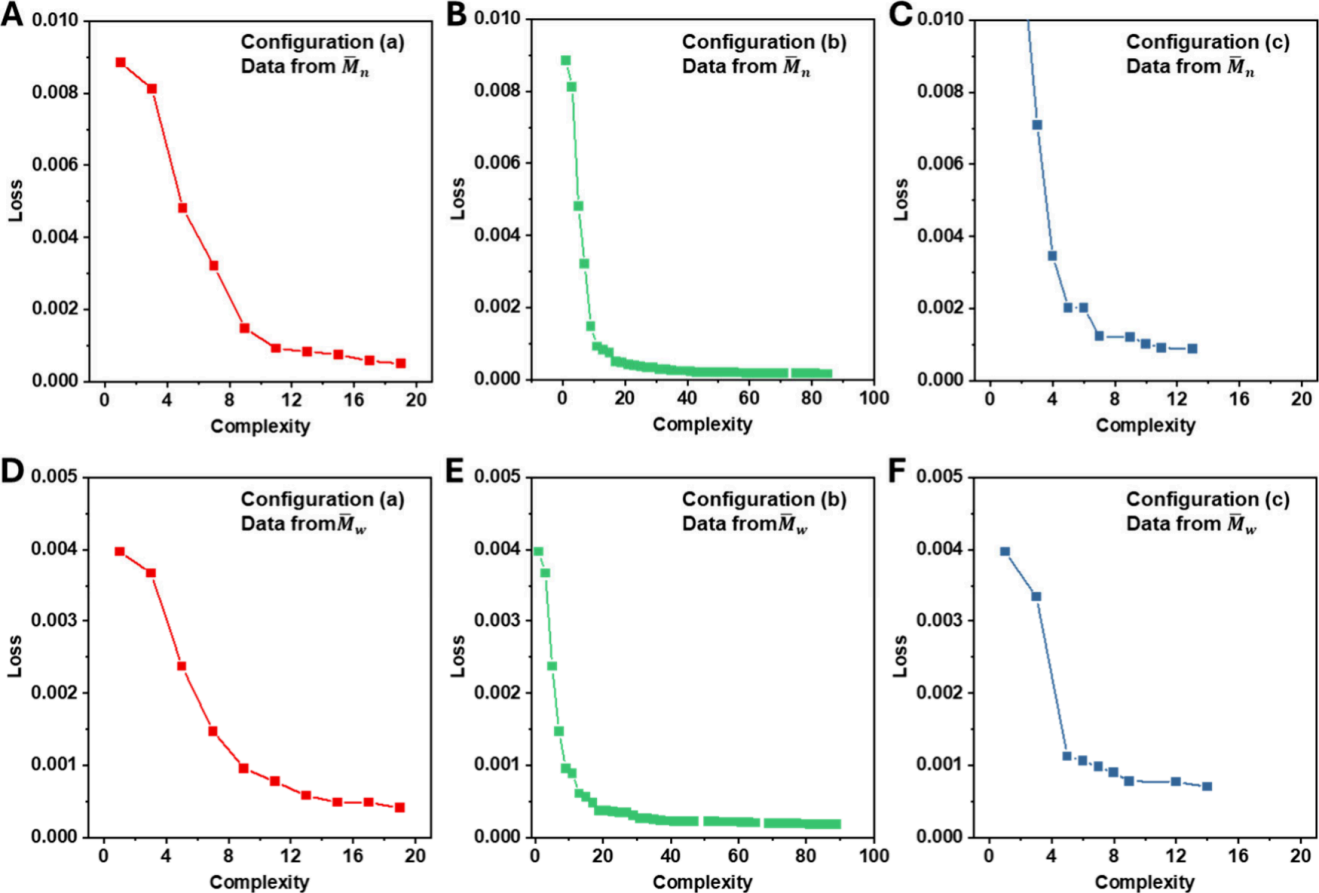
Loss curves of the Pl formulas generated by the symbolic regression program under different levels of formula complexity. (A–C) Loss versus formula complexity when fitting Pl using *M̅*_*n*_-derived input data under three configurations: (A) Configuration (a): operators + , -, *, /, max complexity = 20; (B) Configuration (b): operators + , -, *, /, max complexity = 100; (C) Configuration (c): operators + , -, *, /, exponentiation, square, power, square root, logarithm, and cube, max complexity = 100. (D–F) Corresponding results when fitting Pl using *M̅*_*w*_-derived input data under the same configurations: (D) Configuration (a), (E) Configuration (b), (F) Configuration (c).

In contrast, configuration (c), which included more advanced mathematical functions such as square, power, square root, logarithm, and cube, enabled faster and more stable Loss reduction and convergence as formula complexity increased (Figure 2C). The optimal formula (highlighted in Table S3) achieved a comparable fit to the previous configurations (Figure S8C vs S8A and S8B), but most importantly, it produced smooth and physically reasonable Pl curves across different concentrations and conversion levels (Figure S9C), suggesting it is a promising candidate formula.

As a comparison, fitting performance was evaluated using Pl values derived from *M̅*_*w*_ as input data. The results (Figure 2D–2F vs Figure 2A–2C) showed that *M̅*_*w*_-based data further improved the Loss. The best-fitting formulas (Tables S4–S6 and Figures S10A–S10C) demonstrated excellent agreement with the experimental data. Moreover, when used to generate Pl curves under different concentrations and conversions, configurations (a) and (c) yielded smooth and consistent predictions (Figures S11A, S11C), reinforcing the robustness and generalizability of the formulas.

### Construction of Pc Formulas Using Different Configurations and Input Data

Since Pc is directly related to Pl through a simple expression (Equation 3), a reliable formula for Pc can also be used to derive other cyclization-related equations. Therefore, we further attempted symbolic regression modeling for Pc. The same three hyperparameter configurations were employed as in the Pl fitting: (a) operators: +, -, *, /, max complexity = 20; (b) operators: +, -, *, /, max complexity = 100; (c) operators: +, -, *, /, exponentiation, square, power, square root, logarithm, cube, max complexity = 100. As before, Pc values derived from *M̅*_*n*_ and *M̅*_*w*_ were used separately as input data for the fitting. The resulting formulas under varying complexities are presented in Tables S7–S12.

Regardless of whether *M̅*_*n*_ or *M̅*_*w*_ was used, the fitting trends for Pc closely mirrored those observed for Pl. Specifically, configuration (c), which included advanced mathematical functions, consistently achieved lower Loss values at lower complexity compared to configurations (a) and (b), which used only basic arithmetic operations. However, as formula complexity increased, the final performance of (c) did not surpass that of (a) or (b) (Figure 3C vs [Fig fig3]A, 3B; Figure 3F vs [Fig fig3]D, 3E). When comparing the predictions of the optimal formulas against the actual values, no significant differences in fitting quality were observed (Figures S12, S14). Finally, the optimal formulas were applied to compute Pc values across different reaction concentrations and conversion levels (Figures S13, S15). These results showed that regardless of the input data source, configuration (b) consistently exhibited overfitting, as indicated by the appearance of outliers in Figures S13B, S15B. In contrast, configurations (a) and (c) produced smooth and physically reasonable prediction curves.

**Figure 3:**
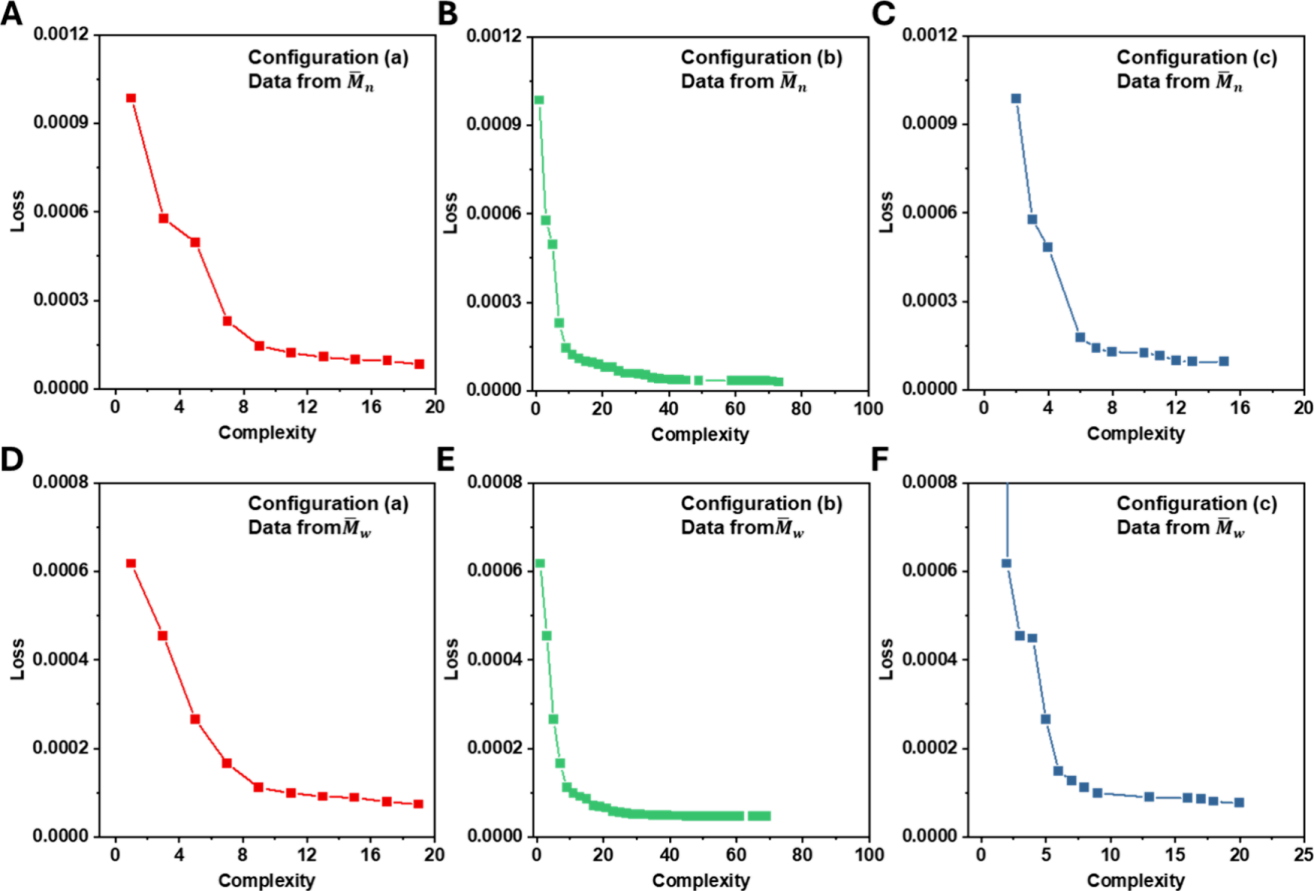
Loss curves of the Pc formulas generated by the symbolic regression program under different levels of formula complexity. (A–C) Loss versus formula complexity when fitting Pc using *M̅*_*n*_-derived input data under three configurations: (A) Configuration (a): operators +, -, *, /, max complexity = 20; (B) Configuration (b): operators +, -, *, /, max complexity = 100; (C) Configuration (c): operators +, -, *, /, exponentiation, square, power, square root, logarithm, and cube, max complexity = 100. (D–F) Corresponding results when fitting Pc using *M̅*_*w*_-derived input data under the same configurations: (D) Configuration (a), (E) Configuration (b), (F) Configuration (c).

### Selection of the Final Formula

Based on the above results, the final formula was selected from the best-performing Pl or Pc expressions, since either one can serve as a foundational expression from which other key parameters, such as *X̅*_*n*_, *X̅*_*w*_, dispersity (*Đ*), and the cyclization probability (CP) at different conversions, can be derived. Among all tested configurations, configuration (b) consistently exhibited overfitting issues (Figures S9B, S11B, S13B, and S15B), while configuration (a) consistently produced better fits than configuration (c), achieving lower Loss values. However, the combination *M̅*_*n*_ + (a) also showed signs of overfitting (Figure S9A). Therefore, the final selection was narrowed down to three candidates: Pl from *M̅*_*w*_ + (a), Pc from *M̅*_*n*_ + (a), and Pc from *M̅*_*w*_ + (a). These three formulas exhibited similar Loss values and formula complexity, making it difficult to determine the best one based solely on fitting performance. Because each formula was derived using only *M̅*_*n*_ or *M̅*_*w*_ data, there was a concern that a given formula might perform poorly when predicting the other metric. To address this, each formula was tested by substituting into the following theoretical expressions and comparing the predicted *M̅*_*n*_ and *M̅*_*w*_ values with the actual experimental data:

10 $${\mathrm{M̅}}_{n}=\frac{1}{1-Pl}{\mathrm{M̅}}_{0}$$

11 $${\mathrm{M̅}}_{w}=\frac{1+Pl}{1-Pl}{\mathrm{M̅}}_{0}$$

12 $${\mathrm{M̅}}_{n}=\frac{1}{1-P+Pc}{\mathrm{M̅}}_{0}$$

13 $${\mathrm{M̅}}_{w}=\frac{1+P-Pc}{1-P+Pc}{\mathrm{M̅}}_{0}$$

As shown in Figure 4, Pc derived from *M̅*_*n*_ + (a) yielded the most accurate overall results compared to the other two candidates. The predicted *M̅*_*n*_ values (Figure 4A) and *M̅*_*w*_ values (Figure 4D) both closely matched the experimental data. In contrast, Pc from *M̅*_*w*_ + (a), although producing good agreement with *M̅*_*w*_ (Figure 4E), significantly overestimated *M̅*_*n*_ for the 10%, 20%, and 30% w/w cases (Figure 4B). Similarly, Pl from *M̅*_*w*_ + (a) also matched *M̅*_*w*_ well (Figure 4F), but noticeably overestimated *M̅*_*n*_ for the 5%, 10%, and 20% w/w cases (Figure 4C). Taken together, these results suggest that Pc derived from *M̅*_*n*_ + (a) offers the best agreement with experimental data and was therefore selected as the final expression (Equation 14).

**Figure 4:**
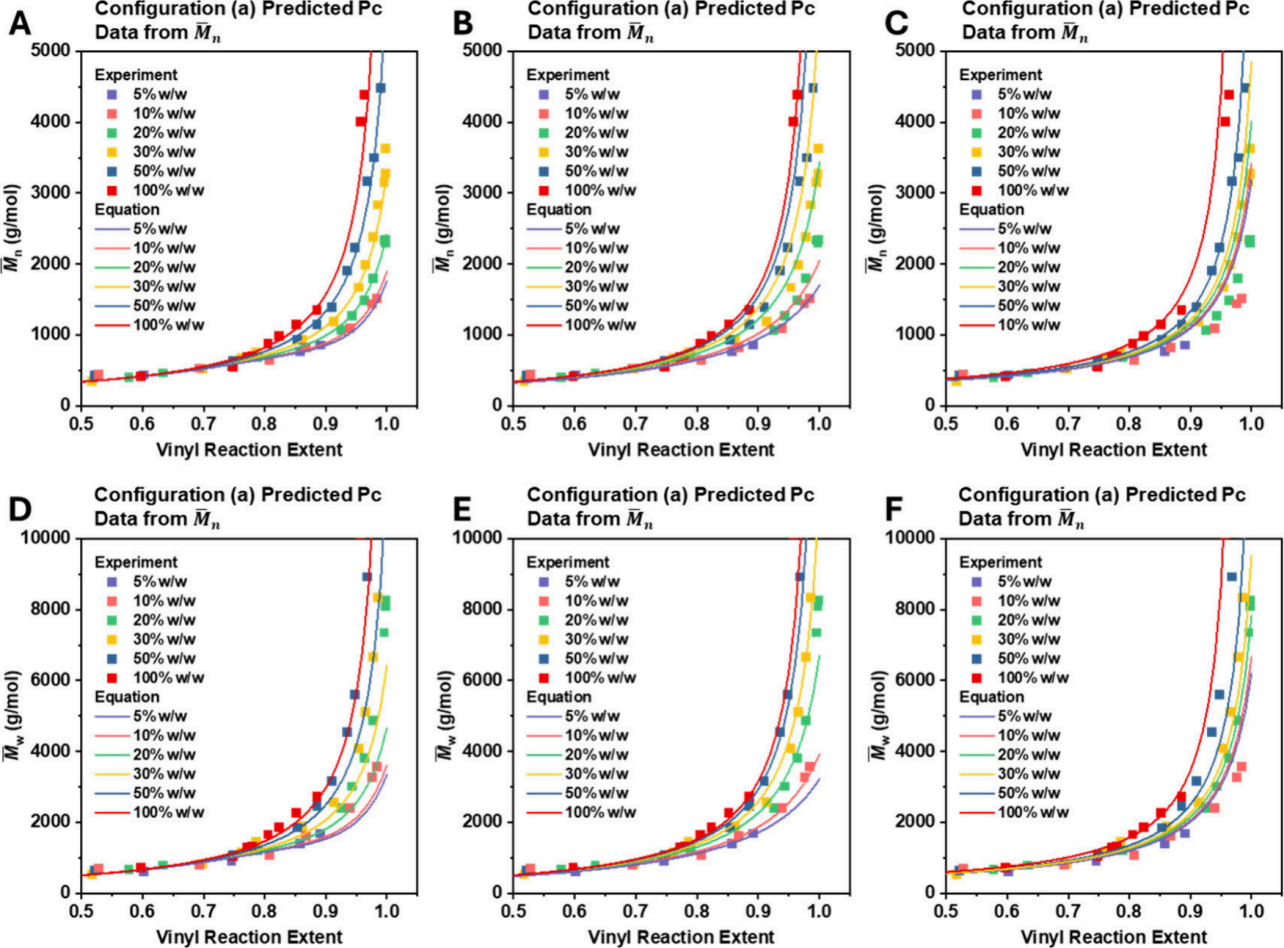
Comparison between calculated and experimental values of *M̅*_*n*_ and *M̅*_*w*_ using different Pc and Pl formulas. (A) *M̅*_*n*_ calculated using the Pc formula derived from *M̅*_*n*_ input data. (B) *M̅*_*n*_ calculated using the Pc formula derived from *M̅*_*w*_ input data. (C) *M̅*_*n*_ calculated using the Pl formula derived from *M̅*_*w*_ input data. (D) *M̅*_*w*_ calculated using the Pc formula derived from *M̅*_*n*_ input data. (E) *M̅*_*w*_ calculated using the Pc formula derived from *M̅*_*w*_ input data. (F) *M̅*_*w*_ calculated using the Pl formula derived from *M̅*_*w*_ input data.

Based on the result of Equation 14, substituting it into Equation 3 allows the derivation of Pl, as shown in Equation 15. Further substituting Equation 15 into Equations 4 and 5 yields expressions for the *X̅*_*n*_ (Equation 16) and the *X̅*_*w*_ (Equation 17) that account for cyclization. From these, the dispersity *Đ* can be calculated as Equation 18. Notably, by differentiating Equation 14, the instantaneous cyclization probability as a function of conversion can be obtained, denoted as Equation 19.

$$Pc=\frac{0.019185985P^{2}}{P^{2}c-0.57804155P+0.6386159}$$ 14

15 $$Pl=P-\frac{0.019185985P^{2}}{P^{2}c-0.57804155P+0.6386159}$$

16 $${\mathrm{X̅}}_{n}=\frac{1}{1-\left(P-\frac{0.019185985}{c+\frac{0.6386159-0.57804155P}{P^{2}}}\right)}$$

17 $${\mathrm{X̅}}_{w}=\frac{1+P-\frac{0.019185985P^{2}}{P^{2}c-0.57804155P+0.6386159}}{1-\left(P-\frac{0.019185985P^{2}}{P^{2}c-0.57804155P+0.6386159}\right)}$$

18 $$Đ=\frac{{\mathrm{X̅}}_{w}}{{\mathrm{X̅}}_{n}}=1+P-\frac{0.019185985P^{2}}{P^{2}c-0.57804155P+0.6386159}$$

19 $$CP=0.019185985\frac{\left(-0.57804155P+1.2772318\right)}{P^{3}{\left(c+\frac{-0.57804155+0.6386159}{P^{2}}\right)}^{2}}$$

where c represents the reaction concentration (w/v).

At this point, closed-form expressions for Pc, Pl, cyclization probability (CP), *X̅*_*n*_, *X̅*_*w*_, and *Đ*, all accounting for different experimental concentrations, have been successfully obtained. The results calculated using these formulas across various values of c and P are shown in Figure 5. As illustrated in Figure 5A, at relatively low concentrations (<50% w/w), the cyclization probability CP increases continuously with conversion, and the lower the concentration, the more pronounced the increase. At extremely dilute conditions (<5% w/w), CP can even exceed 0.5 in the later stages of reaction, high enough to significantly suppress chain growth. In contrast, at high concentrations (>50% w/w), CP initially increases with conversion but then decreases at higher conversions, approaching zero. This may be attributed to increased viscosity in the late stages of polymerization, which hinders intramolecular chain-end collisions. These trends give rise to several resulting phenomena: The lower the reaction concentration, the more conversion is consumed by cyclization at the same overall reaction progress (Figure 5B). Conversely, less conversion is consumed by linear growth (Figure 5C). As a result, *X̅*_*n*_, *X̅*_*w*_, and *Đ* decrease with decreasing concentration (Figures 5D, 5E, 5F).

**Figure 5:**
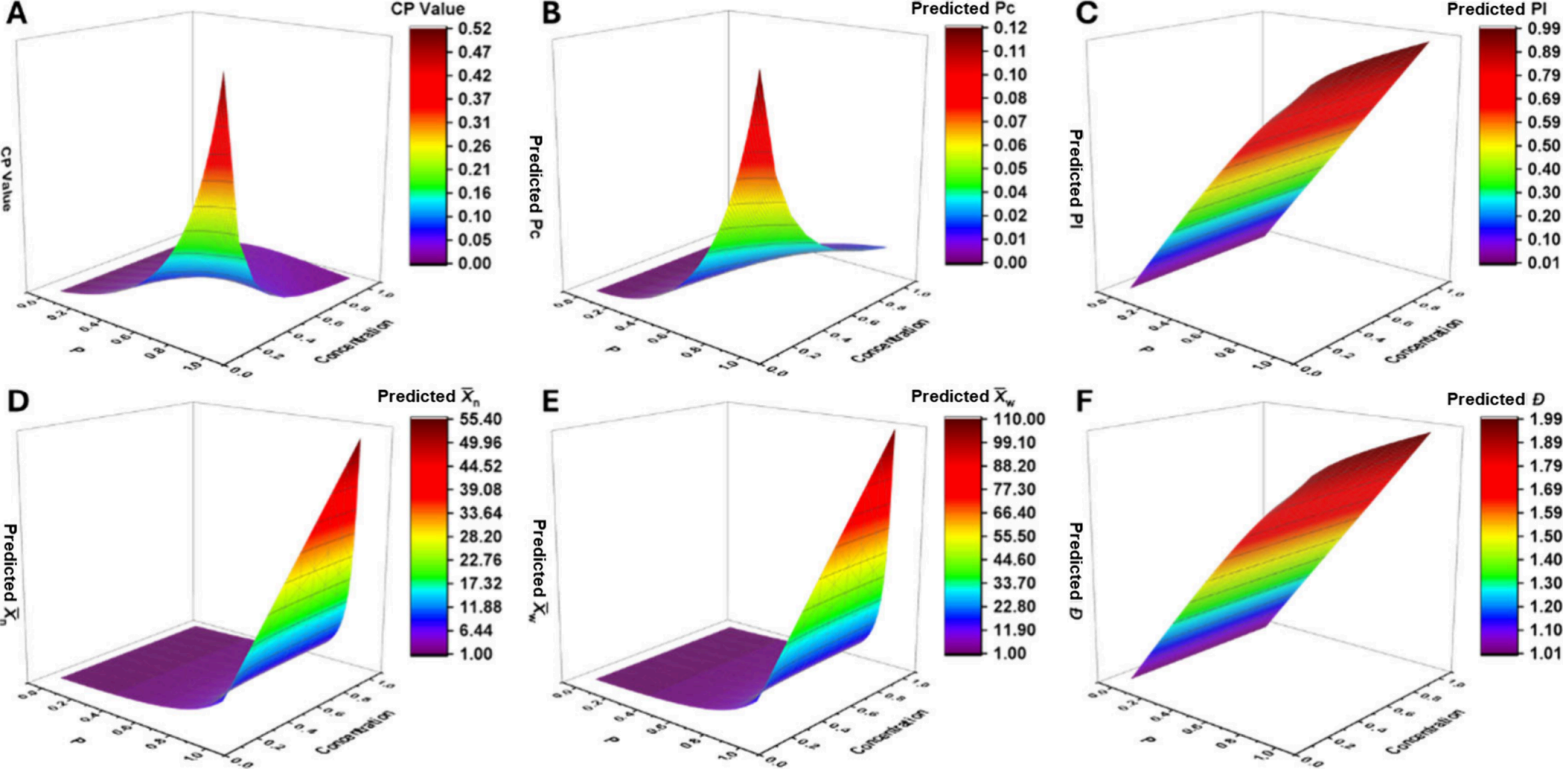
Predicted values from the new cyclization-inclusive formulas across different experimental concentrations. (A) Cyclization probability (CP) at various reaction concentrations and conversion levels, calculated from Equation 19. (B) Pc values at different reaction concentrations and conversions, calculated from Equation 14. (C) Pl values at different reaction concentrations and conversions, calculated from Equation 15. (D) *X̅*_*n*_ calculated from Equation 16. (E) *X̅*_*w*_ calculated from Equation 17. (F) *Đ* calculated from Equation 18.

## Conclusion

This study presents a new theoretical framework for quantifying intramolecular cyclization in linear step-growth polymerizations across varying reaction concentrations. Through a reverse-engineering approach rooted in experimental data and symbolic regression, we have obtained concise and generalizable equations that can describe the extent of cyclization (Pc), linear propagation (Pl), polymerization degree (*X̅*_*n*_, *X̅*_*w*_), dispersity (*Đ*), and the instantaneous cyclization probability (CP). Although this work does not provide a universal formula to describe cyclization reactions across all systems, it offers a practical toolkit by introducing dynamic, condition-sensitive descriptors. This lays the groundwork for future studies on topology-driven polymer properties and for the rational design of polymer structures under realistic reaction conditions, thereby taking a step toward improving classical polymerization theory.

## Supplementary Material



## Notes

Detailed experimental procedures, GPC trace measurements, NMR spectra measurements, predicted Pl and Pc values at different conditions, and tables of Pl and Pc formulas at different levels of complexity (PDF)
