# Supplementary material for: Cyclization in Linear Step-Growth Polymerizations
Source: Macromolecules. 2025 Sep 11;58(18):9653–9. doi: 10.1021/acs.macromol.5c00980 (PMC12462237; doi:10.1021/acs.macromol.5c00980)
Supplement: Supplementary file 1 [file ma5c00980_si_001.pdf]

Supporting Information

Cyclization in linear step-growth polymerizations

Yinghao Li,[†] Jing Lyu*,[†] and Wenxin Wang*,[†,‡]

[†]*Charles Institute of Dermatology, School of Medicine, University College Dublin, Dublin 4, Ireland*

[‡]*Institute of Precision Medicine (AUST-IPM), Anhui University of Science and Technology, Huainan, China*

1. Materials

1,4-butanediol diacrylate (BDA), and N,N′-Dimethyl-1,6-hexanediamine (DHD) were purchased from Sigma-Aldrich. Lithium bromide (LiBr) for GPC measurements was purchased from Sigma-Aldrich. Dichloromethane (DCM), dimethylformamide (DMF) were purchased from Fisher Scientific. Deuterated chloroform ($CDCl_3$) was purchased from Sigma-Aldrich.

2. Polymer Synthesis

Polymers were synthesized through a Michael addition reaction. Specifically, 1,4-butanediol diacrylate (1.98 g) and N,N′-Dimethyl-1,6-hexanediamine (1.78 g) were dissolved into different weight/weight (w/w) with DCM. Then the reactions were carried out at 20 ^{o}C metal bath. Agilent 1260 Infinite gel permeation chromatography (GPC) and nuclear magnetic resonance (NMR) were used to monitor the reaction.

3. Polymer Characterization

An Agilent 1260 Infinite gel permeation chromatography (GPC) equipped with a refractive index detector (RI), was used to monitor the change of functional groups conversion rate, the weight-average molecular weight ($\overline{M}_w$), number-average molecular weight ($\overline{M}_n$), and polydispersity (*Đ*). For GPC measurement, 100 μl of the reaction mixture was taken and diluted in 1ml DMF, and filtered through a 0.45 μm filter. DMF with 0.1% LiBr was utilized to elute the GPC columns (Polar Gel-M, 7.5×300 mm, two in series) at a flow rate of 1ml/min at 60 ^{o}C. Linear poly(methyl methacrylate) (PMMA) standards were used for the calibration of the GPC columns. Meanwhile, the conversion rates and chemical compositions were monitored with ^{1}H

NMR on a 400 MHz Varian Inova spectrometer. The samples were reported in parts per million (ppm) relative to the solvent $CDCl_3$ (7.23 ppm) or internal control (tetramethylsilane 0.00 ppm).

4. Experiment Data

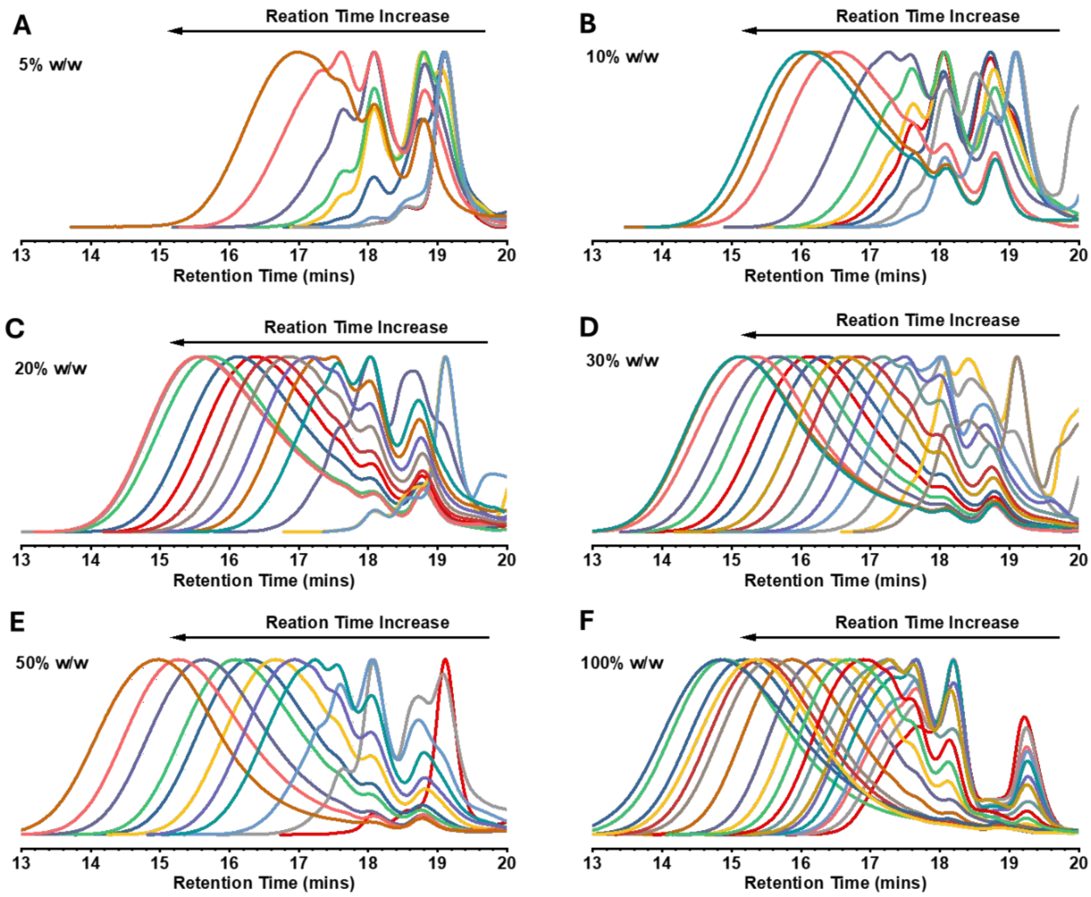

Figure S1. GPC traces of poly(beta-amino ester)s synthesized at different concentrations.

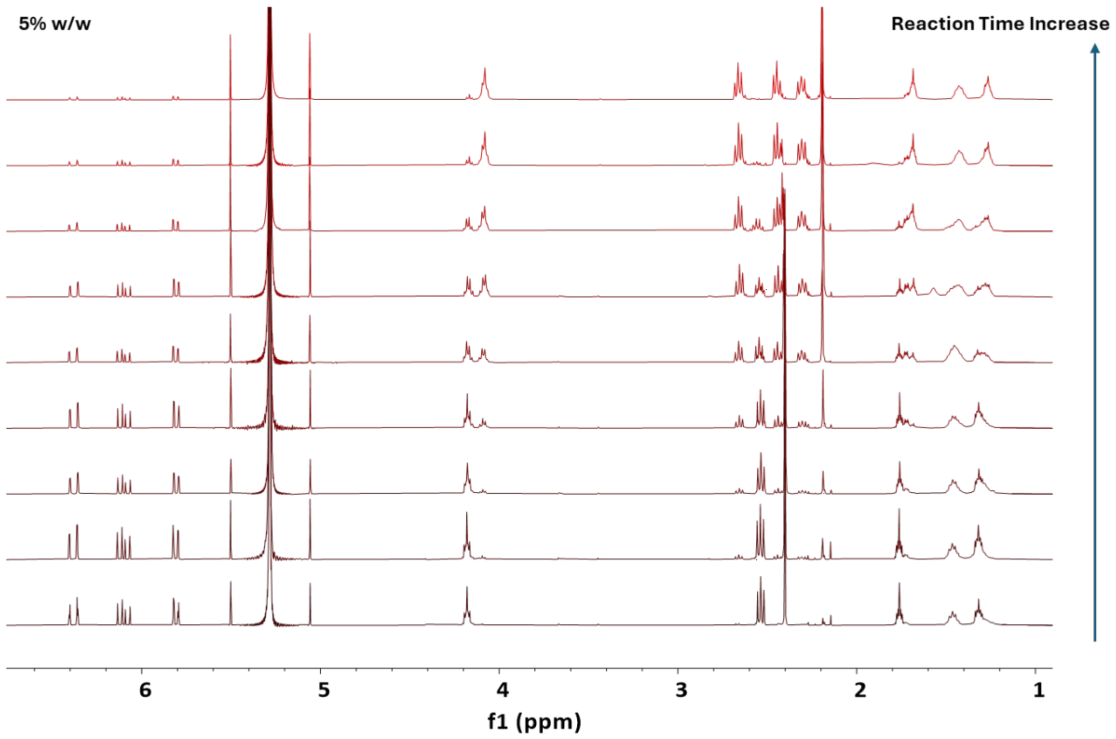

Figure S2. Different time point NMR spectra of poly(beta-amino ester)s synthesized at 5% w/w.

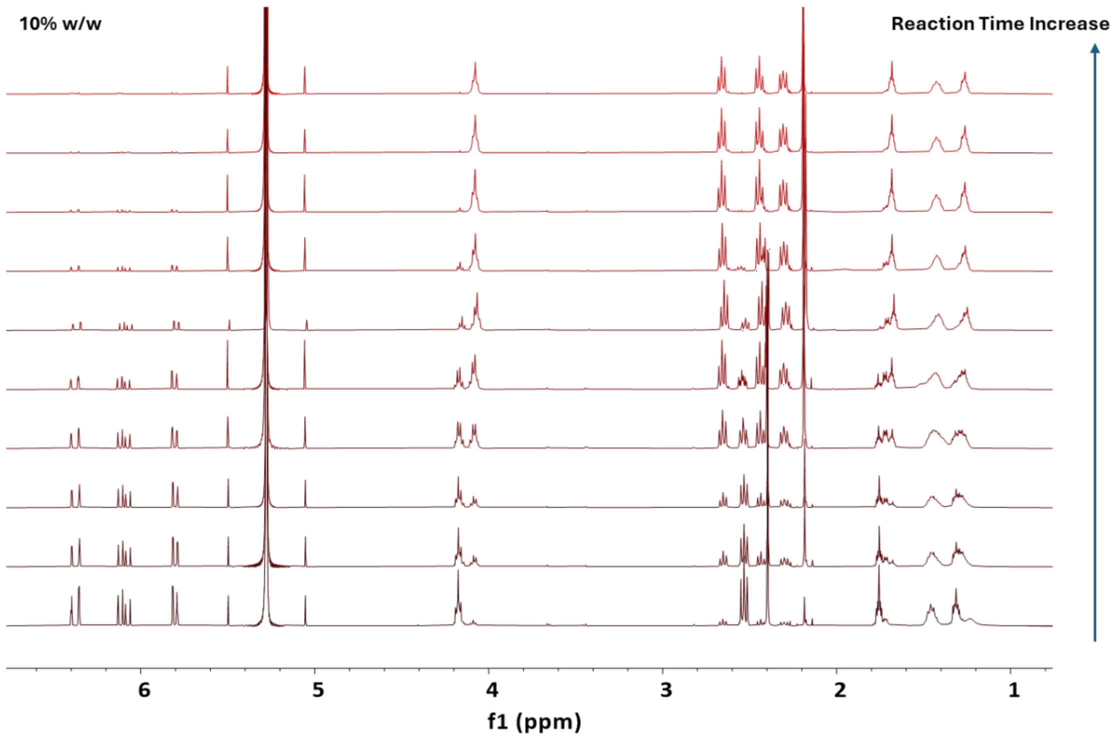

Figure S3. Different time point NMR spectra of poly(beta-amino ester)s synthesized at 10% w/w.

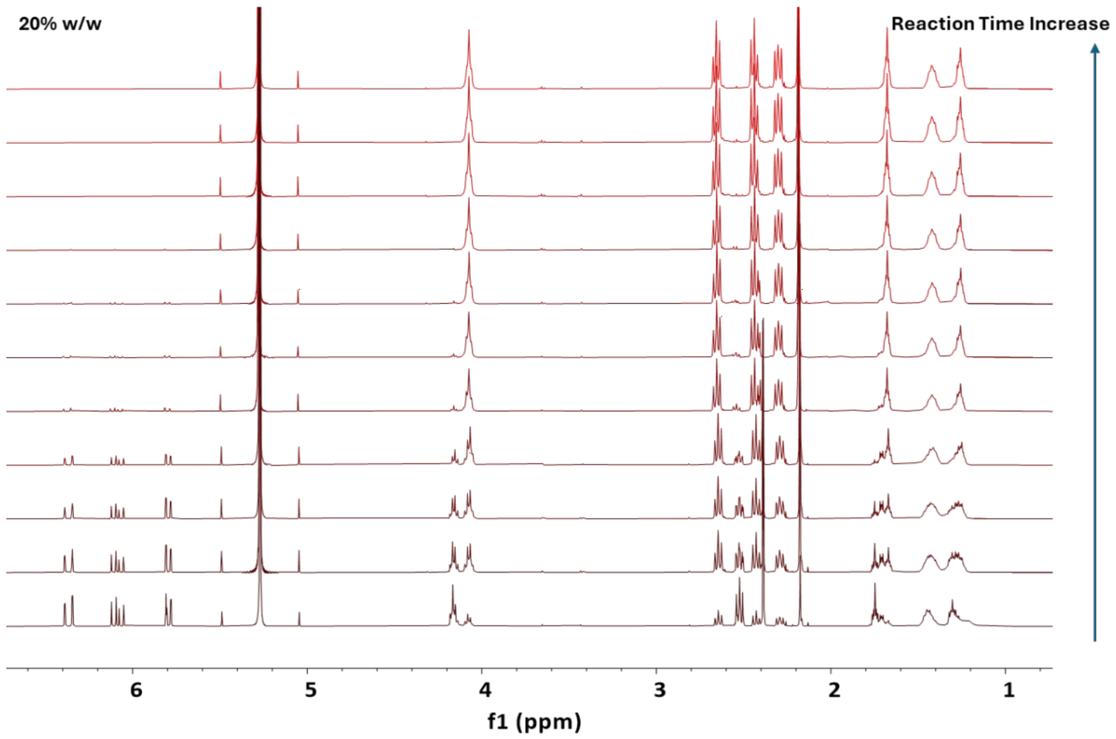

Figure S4. Different time point NMR spectra of poly(beta-amino ester)s synthesized at 20% w/w.

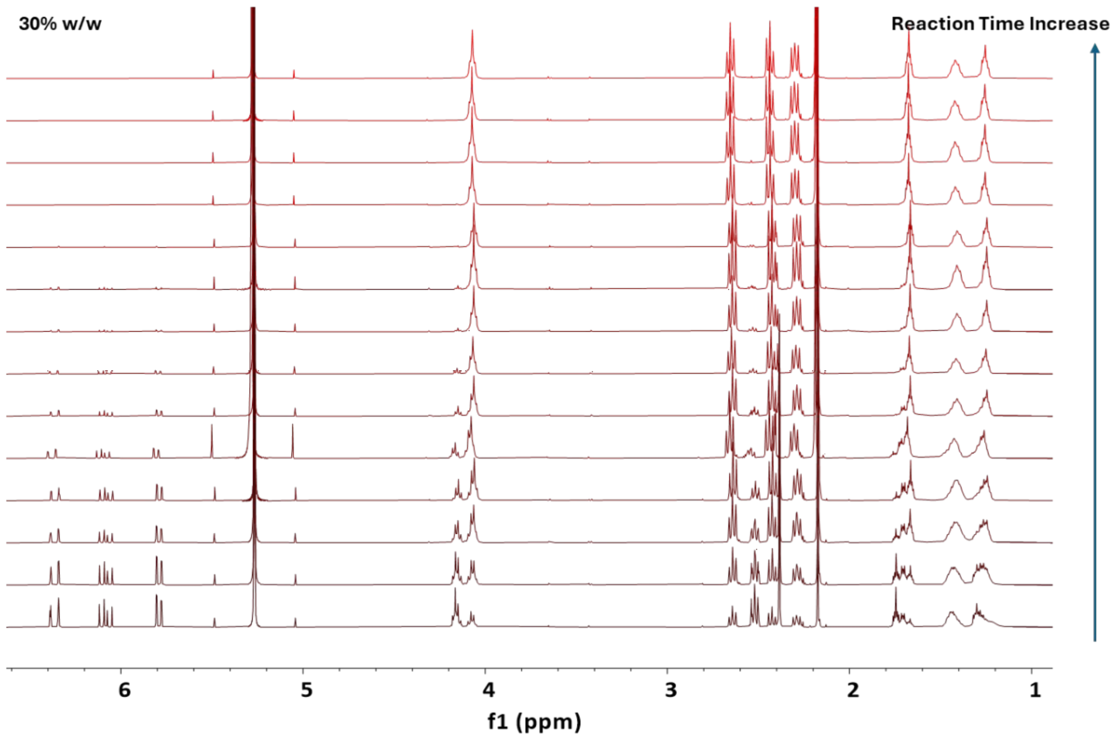

Figure S5. Different time point NMR spectra of poly(beta-amino ester)s synthesized at 30% w/w.

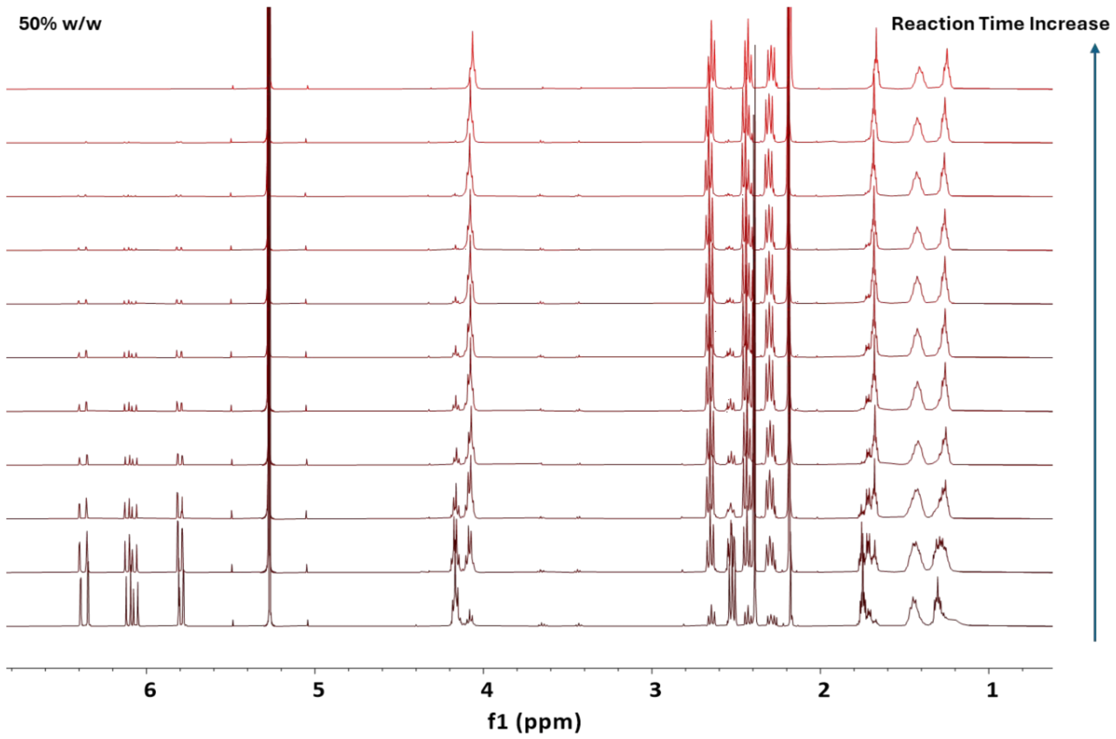

Figure S6. Different time point NMR spectra of poly(beta-amino ester)s synthesized at 50% w/w.

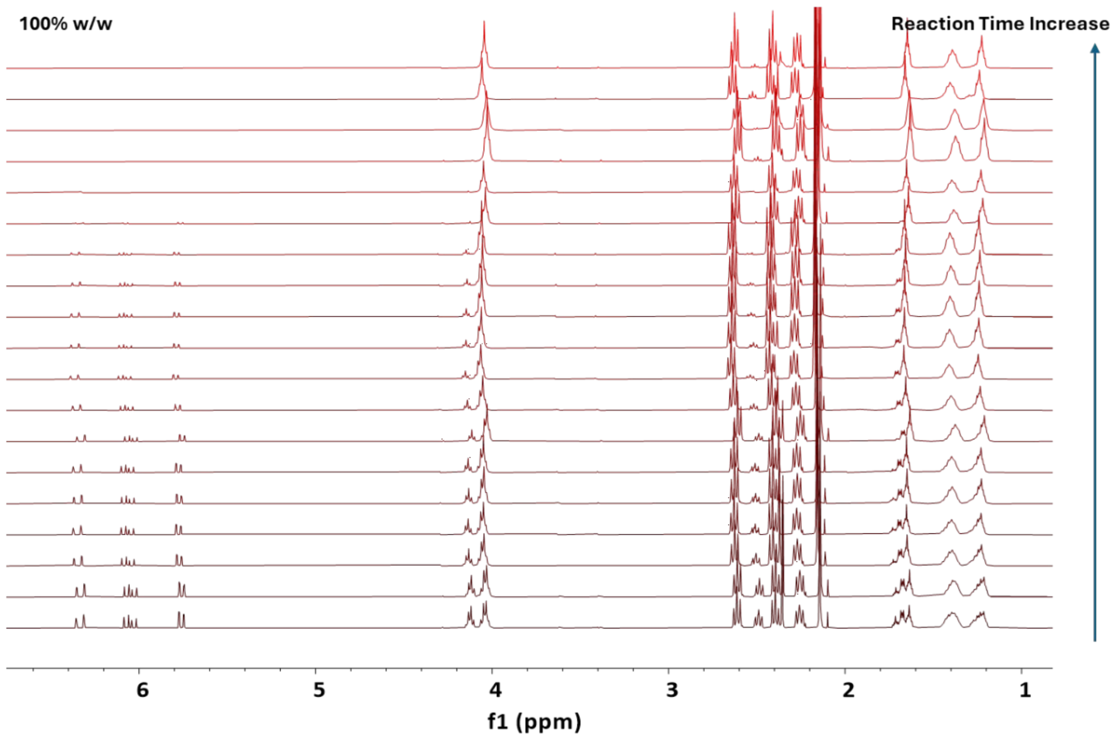

Figure S7. Different time point NMR spectra of poly(beta-amino ester)s synthesized at 100% w/w.

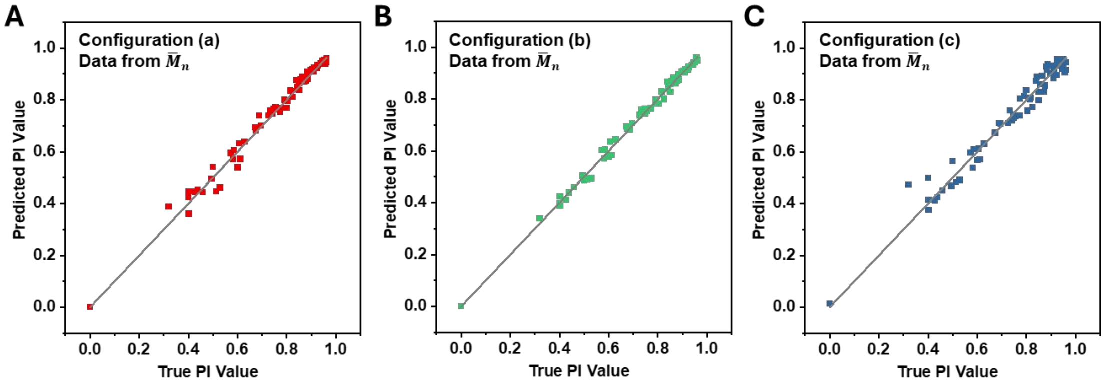

Figure S8. The comparison of predicted Pl value and true Pl value using configuration (A)(a), (B)(b), (C)(c) and $\overline{M}_n$ as input data.

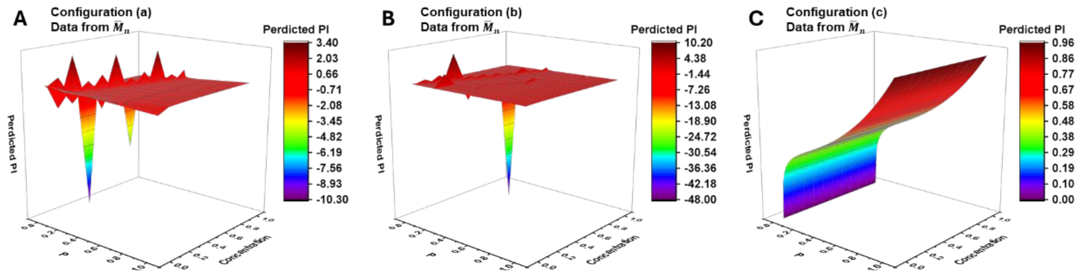

Figure S9. Predicted PI values at different reaction concentrations and conversions using $\overline{M}_n$ as input data. (A) Results from configuration (a); (B) Results from configuration (b); (C) Results from configuration (c).

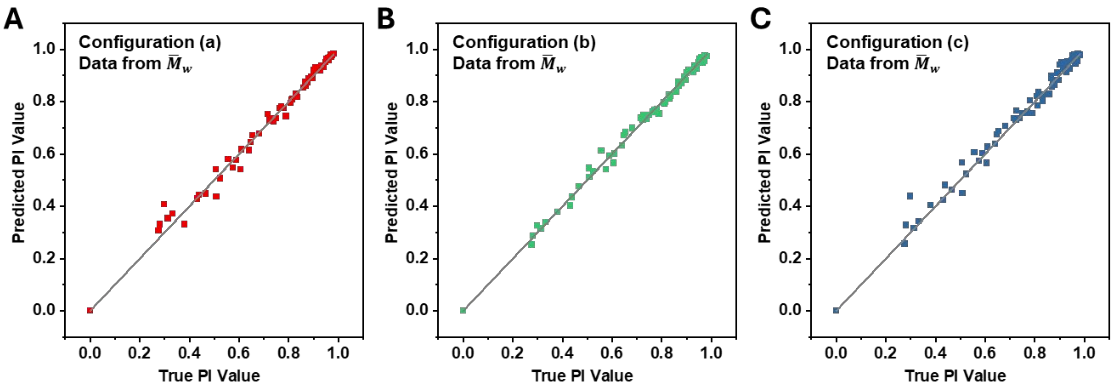

Figure S10. The comparison of predicted Pl value and true Pl value using configuration (A)(a), (B)(b), (C)(c) and $\overline{M}_w$ as input data.

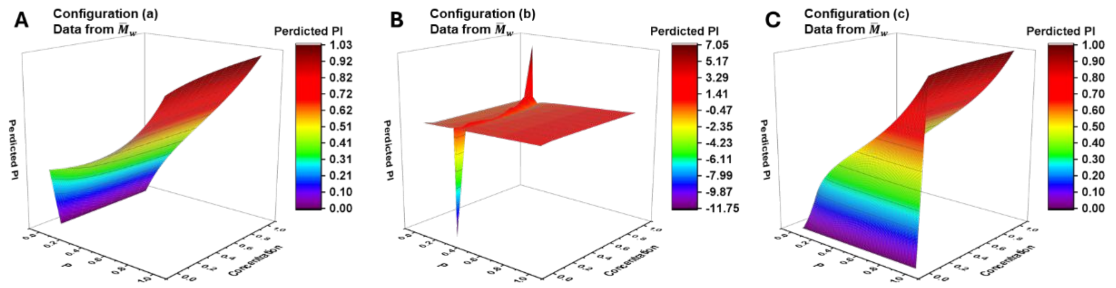

Figure S11. Predicted PI values at different reaction concentrations and conversions using $\overline{M}_w$ as input data. (A) Results from configuration (a); (B) Results from configuration (b); (C) Results from configuration (c).

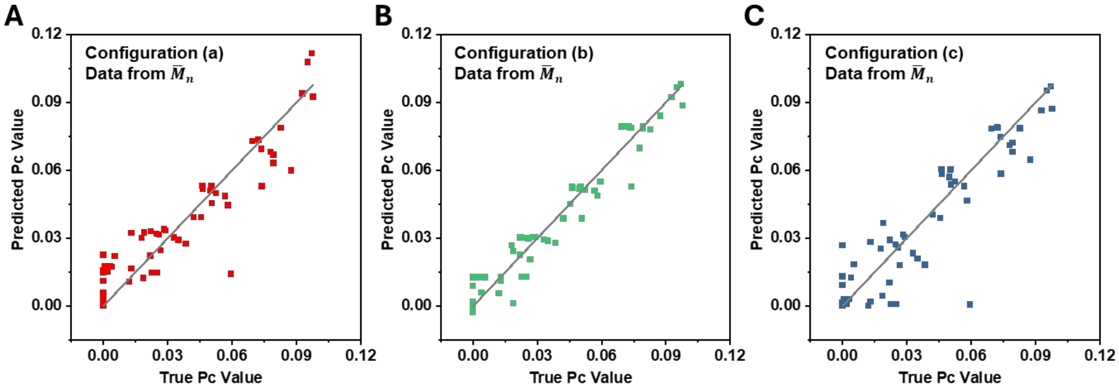

Figure S12. The comparison of predicted Pc value and true Pc value using configuration (A)(a), (B)(b), (C)(c) and $\overline{M}_n$ as input data.

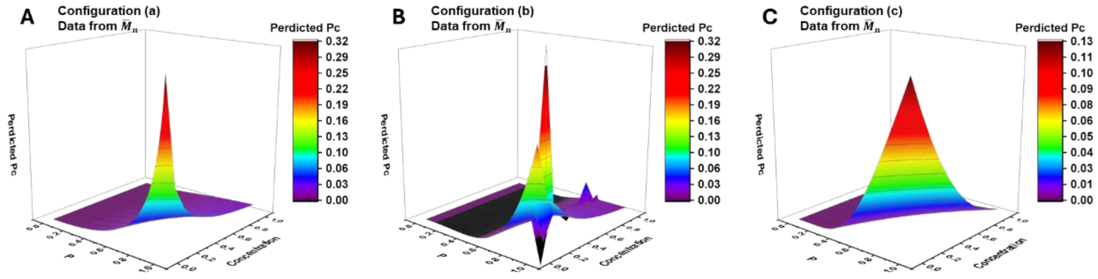

Figure S13. Predicted Pc values at different reaction concentrations and conversions using $\overline{M}_n$ as input data. (A) Results from configuration (a); (B) Results from configuration (b); (C) Results from configuration (c).

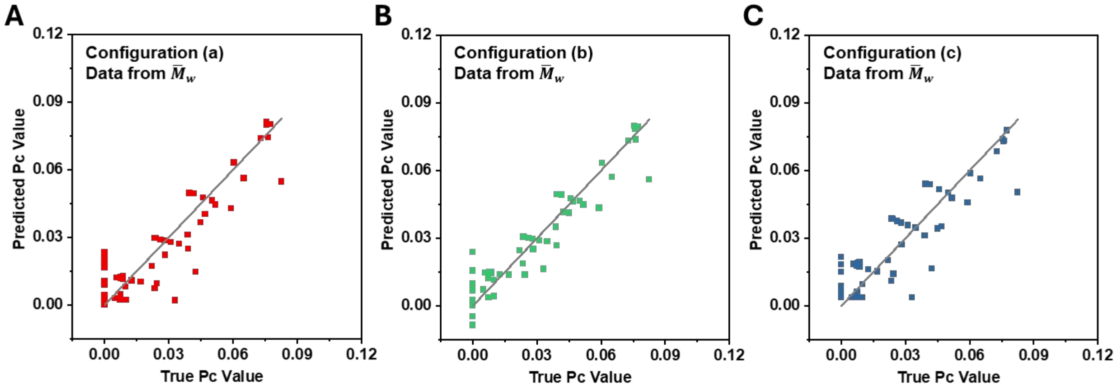

Figure S14. The comparison of predicted Pc value and true Pc value using configuration (A)(a), (B)(b), (C)(c) and $\overline{M}_w$ as input data.

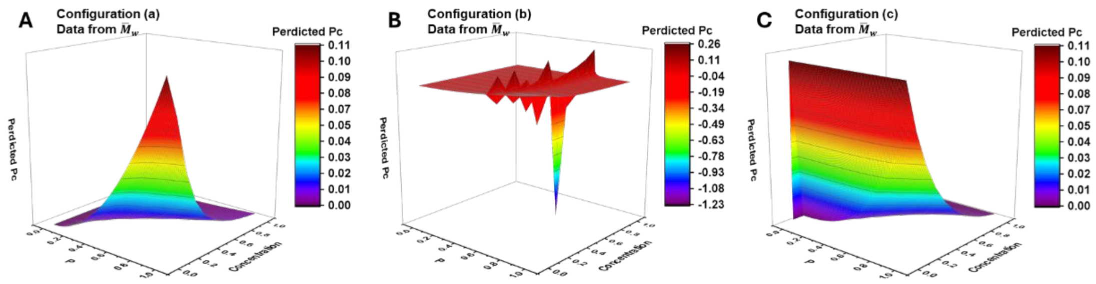

Figure S15. Predicted Pc values at different reaction concentrations and conversions using $\overline{M}_w$ as input data. (A) Results from configuration (a); (B) Results from configuration (b); (C) Results from configuration (c).

Table S1. Pl formulas at different levels of complexity using configuration (a), with $\overline{M}_n$ as input data.

Complexity	Loss	Equation
1	0.008844	P
3	0.008121	P * 0.96472925
5	0.004798	0.13563249 - (-0.8036373 * P)
7	0.003199	P / (1.1358163 + (-0.08342156 / P))
9	0.001481	((0.18578605 / (-0.044321038 + P)) + 0.7298192) * P
11	0.000919	(c - -0.7825307) / (1.8496069 + ((c / P) - P))
13	0.000834	(c - -0.7737338) / ((((c * 0.93978864) / P) - P) + 1.8586383)
15	0.000751	(((P + c) * P) - -1.0870016) / (2.3355143 + ((0.69067836 / P) * c))
**17 (Best)**	0.000571	((P - 0.6583282) / ((c * (((c / 0.09020093) / P) + -25.43273)) - 0.6332184)) + P

19	0.000505	P + ((P - 0.66760683) / ((((c / P) * c) / 0.12196716) + (-1.0335814 - (c / 0.053752974))))

Table S2. Pl formulas at different levels of complexity using configuration (b), with $\overline{M}_n$ as input data.

Complexity	Loss	Equation
1	0.008844	P
3	0.008121	0.96472955 * P
5	0.004798	(P * 0.80361843) + 0.13564387
7	0.003199	P / ((-0.08341929 / P) + 1.1357445)
9	0.001481	(P - -0.2103749) / (1.3703723 - (0.06071181 / P))
11	0.000919	(c - -0.78244823) / (((c / P) - P) + 1.8495085)
13	0.000834	(c - -0.7738899) / ((((0.9398827 * c) / P) - P) + 1.8587791)
15	0.000751	((P * (c + P)) - -1.0870874) / (2.3356361 + ((c * 0.6905993) / P))
17	0.000505	P + ((-0.121713646 * (P + -0.6675332)) / (0.12553987 + (c * (2.2688153 - (c / P)))))

19	0.00047	P + (((P + -0.67230296) * -0.030667573) / (0.053596012 + (((2.0630164 - (c / P)) * c) * c)))
21	0.000411	(P + ((-0.019959778 * (P + -0.7091888)) / ((c * ((2.0603716 - (c / P)) * c)) + 0.033169296))) / 1.0229714
23	0.000388	((-0.120542794 * (P + -0.675302)) / (0.12897353 + ((2.119091 - ((c / P) - (0.009486486 / (0.30707085 - P)))) * c))) + P
25	0.000353	((-0.16413566 * (-0.6614729 + P)) / ((c * (((P - (c / P)) + (0.009976826 / (0.30699348 - P))) + 1.8651117)) + 0.2043913)) + P
27	0.000341	P + ((-0.16252446 * (-0.65744466 + P)) / (0.19816257 + ((2.0301783 + (((P * P) - (c / P)) - (0.01017215 / (P - 0.30699348)))) * c)))
29	0.000329	((((-0.6726049 + P) * -0.1027435) / 0.9504077) / (((c + (1.5046821 + ((0.041429624 / (c + (P + -0.49533337))) - (c / P)))) * c) + 0.15185328)) + P
31	0.000283	((-0.16760246 * (P + -0.6675332)) / ((c * (((P - (c / P)) - (0.14672267 / ((((P * P) / c) - 0.47505575) / P))) + 1.9159118)) + 0.20856038)) + P
33	0.000281	((-0.16760246 * (-0.6675332 + P)) / ((c * (((P - ((c - 0.012813502) / P)) - (0.14672267 / ((((P * P) / c) - 0.47505575) / P))) + 1.9050604)) + 0.20856038)) + P

35	0.000257	((-0.16413566 * (-0.65471154 + P)) / ((c * ((((P * (c + P)) - (c / P)) - (P / ((((P / c) * P) - 0.47505575) / 0.20601818))) + 1.9552839)) + 0.20856038)) + P
37	0.000243	((-0.16413566 * (-0.65471154 + P)) / ((c * ((((P * (c + (P * P))) - (c / P)) - (0.14672267 / ((((P / c) * P) - 0.47505575) / c))) + 1.9159118)) + 0.20856038)) + P
39	0.00024	((-0.16413566 * (-0.65471154 + P)) / ((c * ((((P * (c + (P * P))) - (c / P)) - (0.14672267 / (((((P / c) * P) * 1.0127724) - 0.47505575) / c))) + 1.9159118)) + 0.20856038)) + P
41	0.000231	((-0.16413566 * (-0.65471154 + P)) / ((c * ((((P * (c + ((P * P) * P))) - (c / P)) - (0.14672267 / (((((P / c) * P) * 1.0127724) - 0.47505575) / c))) + 1.9159118)) + 0.20856038)) + P
**43 (Best)**	0.000207	P + ((-0.16760246 * (P + (-0.6606533 - (0.0027949093 * (P / (((P * P) * 1.0985149) - c)))))) / ((c * (((P - (c / P)) - (0.14672267 / ((((P / c) * P) - 0.47505575) / P))) + 1.9159118)) + 0.20856038))
45	0.000204	P + ((-0.16760246 * (P + (-0.6606533 - (0.0027949093 * ((P / (((P * P) * 1.0985149) - c)) - -1.7619694))))) / ((c * (((P - (c / P)) - (0.14672267 / ((((P / c) * P) - 0.47505575) / P))) + 1.9159118)) + 0.20856038))
47	0.000202	P + ((-0.16760246 * (P + (-0.6606533 - (0.0027949093 * ((P / (((P * P) * 1.0985149) - c)) - (-1.7619694 - c)))))) / ((c * (((P -

		(c / P)) - (0.14672267 / ((((P / c) * P) - 0.47505575) / P))) + 1.9159118)) + 0.20856038))
49	0.000201	P + ((-0.16760246 * (P + (-0.6606533 - (0.0027949093 * ((P / (((P * P) * 1.0985149) - c)) - (-1.7619694 - (c * 2.2649252))))))) / ((c * (((P - (c / P)) - (0.14672267 / ((((P / c) * P) - 0.47505575) / P))) + 1.9159118)) + 0.20856038))
51	0.000197	P + ((-0.16760246 * (P + (-0.6606533 - (0.0027949093 * ((P / (((P * P) * 1.0985149) - c)) - ((-3.5529482 * 2.2505505) * c)))))) / ((c * (((P - (c / P)) - ((-0.19875133 * -0.7475736) / ((((P / c) * P) - 0.47505575) / P))) + 1.9159118)) + 0.20856038))
53	0.000197	P + ((-0.16760246 * (P + (-0.6606533 - (0.0027949093 * ((P / (((P * P) * 1.0985149) - c)) - ((-3.5529482 * (1.9565051 + c)) * c)))))) / ((c * (((P - (c / P)) - ((-0.19875133 * -0.7475736) / ((((P / c) * P) - 0.47505575) / P))) + 1.9159118)) + 0.20856038))
55	0.000194	P + ((-0.16760246 * (P + (-0.6606533 - (0.0027949093 * ((P / (((P * P) * 1.0985149) - c)) - ((P / ((-0.21865582 + c) * -0.69129974)) * c)))))) / ((c * (((P - (c / P)) - ((-0.19875133 * -0.7475736) / ((((P / c) * P) - 0.47505575) / P))) + 1.9159118)) + 0.20856038))

57	0.000186	P + ((-0.16760246 * (P + (-0.6606533 - (0.0027949093 * ((P / (((P * P) * 1.0985149) - c)) - (((P / ((-0.21865582 + c) * -0.69129974)) * c) - 2.747017)))))) / ((c * (((P - (c / P)) - ((-0.19875133 * -0.7475736) / ((((P / c) * P) - 0.47505575) / P))) + 1.9159118)) + 0.20856038))
59	0.000182	P + ((-0.16760246 * (P + (-0.6606533 - (0.0027949093 * ((P / (((P * P) * 1.0985149) - c)) - (((P / (((-0.21865582 + c) * -0.69129974) * P)) * c) - 2.747017)))))) / ((c * (((P - (c / P)) - ((-0.19875133 * -0.7475736) / ((((P / c) * P) - 0.47505575) / P))) + 1.9159118)) + 0.20856038))
61	0.000181	P + ((-0.16760246 * (P + (-0.6606533 - (0.0027949093 * ((P / (((P * P) * 1.0985149) - c)) - ((((P / (((-0.21865582 + c) * -0.69129974) * P)) * c) - c) - 2.747017)))))) / ((c * (((P - (c / P)) - ((-0.19875133 * -0.7475736) / ((((P / c) * P) - 0.47505575) / P))) + 1.9159118)) + 0.20856038))
63	0.000179	P + ((-0.16760246 * (P + (-0.6606533 - (0.0027949093 * ((P / (((P * P) * 1.0985149) - c)) - ((((P / (((-0.21865582 + c) * -0.69129974) * P)) - (P + 3.270804)) * c) - 2.747017)))))) / ((c * (((P - (c / P)) - ((-0.19875133 * -0.7475736) / ((((P / c) * P) - 0.47505575) / P))) + 1.9159118)) + 0.20856038))
65	0.000179	P + ((-0.16760246 * (P + (-0.6606533 - (0.0027949093 * ((P / (((P * P) * 1.0985149) - c)) - ((((P / (((-0.21865582 + c) * -

		0.6901344) * P)) - ((P + 3.270804) * c)) * c) - 2.747017)))))) / ((c * (((P - (c / P)) - ((-0.19875133 * -0.7475736) / ((((P / c) * P) - 0.47505575) / P))) + 1.9159118)) + 0.20856038))
67	0.000179	P + ((-0.16760246 * (P + (-0.6606533 - (0.0027949093 * ((P / (((P * P) * 1.0985149) - c)) - ((((P / (((-0.21865582 + c) * -0.69129974) * P)) - ((P + (3.270804 + P)) * c)) * c) - 2.747017)))))) / (((((P - (c / P)) - ((-0.19875133 * -0.7475736) / ((((P / c) * P) - 0.47505575) / P))) + 1.9159118) * c) + 0.20856038))
69	0.000177	P + ((-0.16760246 * (P + (-0.6606533 - (0.0027949093 * ((P / (((P * P) * 1.0985149) - c)) - ((((P / (((-0.21865582 + c) * -0.69129974) * P)) - ((P + (3.270804 + P)) * c)) * c) - 2.747017)))))) / (((((P - (c / P)) - ((-0.19875133 * -0.7475736) / (((((P / c) * P) - 0.47505575) / 1.0302898) / P))) + 1.9159118) * c) + 0.20856038))
71	0.000176	P + ((-0.16760246 * (P + (-0.6606533 - (0.0027949093 * ((P / (((P * P) * 1.0985149) - c)) - ((((P / (((-0.21865582 + c) * -0.69129974) * P)) - ((P + ((3.270804 + P) + P)) * c)) * c) - 2.747017)))))) / (((((P - (c / P)) - ((-0.19875133 * -0.7475736) / (((((P / c) * P) - 0.47505575) / 1.0302898) / P))) + 1.9159118) * c) + 0.20856038))

75	0.000171	P + ((-0.16760246 * (P + (-0.6606533 - (0.0027949093 * ((((c / c) / (((P * P) * 1.0985149) - c)) - (((((P / -0.63864434) / (((-0.21865582 + (c / 0.7775933)) * P) * -1.0810535)) - ((1.8903178 / 0.29329985) + 1.9783964)) * (c + c)) / P)) * P))))) / ((c * (((P - (c / P)) - ((-0.19875133 * -0.7475736) / ((((P / c) * P) - 0.47505575) / P))) + 1.9159118)) + 0.20856038))
77	0.00017	P + ((-0.16760246 * (P + (-0.6606533 - (0.0027949093 * ((((c / c) / (((P * P) * 1.0985149) - c)) - (((((((P / -0.63864434) / (((-0.21865582 + (c / 0.7775933)) * P) * -1.2614993)) - 1.8903178) - 0.9741508) + -1.7938637) * ((c + c) + c)) / P)) * P))))) / ((c * (((P - (c / P)) - ((-0.19875133 * -0.7475736) / ((((P / c) * P) - 0.47505575) / P))) + 1.9159118)) + 0.20856038))
79	0.000169	P + ((-0.16760246 * (P + (-0.6606533 - (0.0027949093 * ((((c / c) / (((P * P) * 1.0985149) - c)) - ((((((((P / -0.63864434) / (((-0.21865582 + (c / 0.7775933)) * P) * -1.2614993)) - P) - 1.8903178) - 0.9741508) + -1.7938637) * ((c + c) + c)) / P)) * P))))) / ((c * (((P - (c / P)) - ((-0.19875133 * -0.7475736) / ((((P / c) * P) - 0.47505575) / P))) + 1.9159118)) + 0.20856038))

81	0.000165	P + ((-0.16760246 * (P + (-0.6606533 - (0.0027949093 * (((P + ((c / c) / (((P * P) * 1.0985149) - c))) - (((((((P / -0.63864434) / (((-0.21865582 + (c / 0.7775933)) * (P + -0.1238808)) * -1.2614993)) - 1.8903178) - P) - P) * ((c + c) + c)) / P)) * P))))) / ((c * (((P - (c / P)) - ((-0.19875133 * -0.7475736) / ((((P / c) * P) - 0.47505575) / P))) + 1.9159118)) + 0.20856038))
83	0.00016	P + ((-0.16760246 * (P + (-0.6606533 - (0.0027949093 * (((P + ((c / c) / (((P * P) * 1.0985149) - c))) - ((((((((P / -0.63864434) / (((-0.21865582 + (c / 0.7775933)) * (P + -0.1238808)) * -1.2614993)) - 1.8903178) - P) + -1.7938637) - P) * ((c + c) + c)) / P)) * P))))) / ((c * (((P - (c / P)) - ((-0.19875133 * -0.7475736) / ((((P / c) * P) - 0.47505575) / P))) + 1.9159118)) + 0.20856038))
85	0.000159	P + ((-0.16760246 * (P + (-0.6606533 - (0.0027949093 * (((P + ((c / c) / (((P * P) * 1.0985149) - c))) - ((((((((P / -0.63864434) / ((((-0.21865582 + (c / 0.7775933)) * 1.0627677) * (P + -0.1238808)) * -1.2614993)) - 1.8903178) - P) + -1.7938637) - P) * ((c + c) + c)) / P)) * P))))) / ((c * (((P - (c / P)) - ((-0.19875133 * -0.7475736) / ((((P / c) * P) - 0.47505575) / P))) + 1.9159118)) + 0.20856038))

Table S3. Pl formulas at different levels of complexity using configuration (c), with $\overline{M}_n$ as input data.

Complexity	Loss	Equation
2	0.011946	sqrt(P)
3	0.007091	P ^ 0.78105134
4	0.003451	sqrt(P * 0.7903787)
5	0.002025	P ^ (P + 0.3487362)
6	0.002025	P ^ (cube(0.70387936) + P)
**7 (Best)**	0.001219	(P * 0.966332) ^ (0.31010175 + P)
9	0.001208	(P / (1.0368199 ^ P)) ^ (0.32161734 + P)
10	0.001019	(P / (c ^ cube(-0.2918875))) ^ (P + 0.33718103)
11	0.000908	(P / (1.0619656 ^ (0.9920042 - c))) ^ (0.30702195 + P)
13	0.000884	(P / (1.0096772 ^ (square(cube(P)) / c))) ^ (0.33718103 + P)

Table S4. Pl formulas at different levels of complexity using configuration (a), with $\overline{M}_w$ as input data.

Complexity	Loss	Equation
1	0.003971	P
3	0.003672	P * 0.9773438
5	0.00237	(0.8764658 * P) + 0.08493705
7	0.001471	P / (1.1123444 + (-0.07438592 / P))
9	0.000958	0.85867393 / (1.680936 - (P + (-0.19632438 / P)))
11	0.000773	(c + 0.5981485) / ((1.6176466 + (c / P)) - P)
**13 (Best)**	0.00058	(0.5940123 + (c / 0.92728704)) / ((c / P) + (1.6373243 - P))
15	0.000488	((c / 0.43410245) + 0.6627813) / (((c / 0.4529981) / P) + (1.7308321 - P))
17	0.000487	((c / 0.44476286) + 0.6673782) / ((((c / 0.46437287) - -0.0074369228) / P) + (1.7268518 - P))

19	0.00041	(0.6925561 + P) / (2.7980826 - (((c - (-0.0132324025 / (P * (-0.31669608 + P)))) * 0.13622741) + P))

Table S5. Pl formulas at different levels of complexity using configuration (b), with $\overline{M}_w$ as input data.

Complexity	Loss	Equation
1	0.003971	P
3	0.003672	P * 0.9773438
5	0.00237	(P + 0.09690567) * 0.8764681
7	0.001471	P / (1.1123074 - (0.07437878 / P))
9	0.000958	0.8582408 / ((1.6806475 - P) - (-0.19611779 / P))
11	0.000891	(0.75928646 + P) / ((2.7692606 - P) - (-0.043231722 / P))
13	0.000608	0.7963866 / (((1.6819578 + (c * -0.08011449)) - P) - (-0.1593051 / P))
15	0.000562	0.81171215 / ((0.057589643 / (0.38844967 + c)) + ((1.5817753 - P) - (-0.16274829 / P)))
17	0.000479	0.8232699 / (((1.6906103 - (0.03052536 * (c / (P - 0.31448427)))) - P) - (-0.16982783 / P))

19	0.000372	0.80610293 / (((1.6969248 + (-0.08141264 * (c + (0.024159547 / (P + -0.30843174))))) - P) - (-0.1593051 / P))
21	0.000367	0.8121452 / (((1.6956384 + ((c + (-0.024914803 / (0.10935102 + (c - P)))) * -0.087444894)) - P) - (-0.16646428 / P))
23	0.000362	0.83474743 / ((((-0.09551559 * (((-0.027343856 / ((c - P) + 0.10935102)) + c) + -0.04223233)) + 1.7124788) - P) - (-0.1736571 / P))
25	0.000348	0.80610293 / (((1.6969248 + (((-0.019149289 / (c + -0.6284476)) + (c + (0.021312328 / (P + -0.30843174)))) * -0.08141264)) - P) - (-0.1593051 / P))
27	0.000341	0.80610293 / (((1.6969248 + ((((-0.019149289 / (c + -0.6284476)) + (c + (0.021312328 / (P + -0.30843174)))) + -0.039063904) * -0.08141264)) - P) - (-0.1593051 / P))
29	0.000307	((P + -0.62654173) * ((-0.5063958 * P) / ((c / (1.0395863 - P)) + (P - (c * (((P - c) / (P - 0.30867785)) * 0.18941319)))))) + P
**31 (Best)**	0.000267	((P + -0.62654173) * ((-0.49237034 * P) / ((c / (1.0395863 - P)) + (P - ((c + -0.031646725) * (((P - c) / (P - 0.30867785)) * 0.27788368)))))) + P
33	0.000261	((P + -0.62654173) * ((-0.48982432 * P) / ((c / (1.0395863 - P)) + (P - ((c + -0.057566248) * ((((P - c) / (P - 0.30867785)) * P) / 0.8171877)))))) + P

35	0.000252	((P + -0.62654173) * ((-0.49237034 * P) / ((c / (1.0395863 - P)) + (P - ((c + -0.057566248) * (((((P - c) / (P - 0.30867785)) - P) * P) / 0.8121452)))))) + P
37	0.000233	((P + -0.62654173) * ((-0.49237034 * P) / ((c / (1.0395863 - P)) + (P - ((c + -0.057566248) * (((((P - c) / (P - 0.30867785)) - P) * P) / (1.0395863 - P))))))) + P
39	0.00023	((P + -0.62654173) * (((-0.49237034 * P) * 0.97068226) / ((c / (1.0395863 - P)) + (P - ((c + -0.057566248) * (((((P - c) / (P - 0.30867785)) - P) * P) / (1.0395863 - P))))))) + P
41	0.000228	((P + -0.62654173) * (((-0.49237034 * P) * 0.96627146) / ((c / (1.0395863 - P)) + ((P + -0.0051103625) - ((c + -0.057566248) * (((((P - c) / (P - 0.30867785)) - P) * P) / (1.0395863 - P))))))) + P
43	0.000223	((P + -0.62654173) * (((-0.49237034 * P) * 0.88311553) / ((c / (1.0395863 - P)) + (((P + -0.032136507) - ((c + -0.057566248) * (((((P - c) / (P - 0.30867785)) - P) * P) / (1.0395863 - P)))) / 0.93905437)))) + P
45	0.000221	((P + -0.62654173) * (((-0.49237034 * P) * 0.88311553) / ((c / (1.0395863 - P)) + (((P + -0.032136507) - ((c + -0.057566248) * (((((P - c) / (P - 0.30867785)) - (P / 1.1117432)) * P) / (1.0395863 - P)))) / 0.93905437)))) + P

47	0.00022	((P + -0.62654173) * (((-0.49237034 * P) * 0.88311553) / ((c / (1.0395863 - P)) + (((P + -0.032136507) - ((c + (-0.057566248 * 0.9942805)) * (((((P - c) / (P - 0.30867785)) - (P / 1.1117432)) * P) / (1.0395863 - P)))) / 0.93905437)))) + P
51	0.000219	(((P + -0.62939286) - -0.007309799) * ((-0.49237034 * P) / ((c / (1.0395863 - P)) + (P - ((c + -0.057566248) * ((((((P - c) / (P - 0.30867785)) - (((c + P) / (1.0395863 - P)) * (c + (c + -0.40399972)))) * 1.126988) * P) / 0.8171877)))))) + P
53	0.000218	((((P + -0.62939286) - -0.007309799) - -0.007309799) * ((-0.49237034 * P) / ((c / (1.0395863 - P)) + (P - ((c + -0.057566248) * ((((((P - c) / (P - 0.30867785)) - (((c + P) / (1.0395863 - P)) * (c + (c + -0.37225553)))) * 1.126988) * P) / 0.8171877)))))) + P
55	0.000216	((((P + -0.62939286) - -0.007309799) - -0.007309799) * ((-0.49237034 * P) / ((c / (1.0395863 - P)) + (P - ((c + -0.057566248) * ((((((P - c) / (P - 0.30867785)) - (((P + c) / (1.0395863 - P)) * (c + ((c + -0.37225553) * P)))) * 1.126988) * P) / 0.8171877)))))) + P
57	0.000216	((((P + -0.62939286) - -0.007309799) - -0.007309799) * ((-0.49237034 * P) / ((c / (1.0395863 - P)) + (P - ((c + -0.057566248) * ((((((P - c) / (P - 0.30867785)) - (((P + c) /

		(1.0395863 - P)) * (c + (((c + -0.37225553) * P) * P)))) * 1.126988) * P) / 0.8171877)))))) + P
59	0.000215	((((P + -0.62939286) - -0.007309799) - -0.007309799) * ((-0.49237034 * P) / ((c / (1.0395863 - P)) + (P - ((c + -0.057566248) * ((((((P - c) / (P - 0.30867785)) - (((P + c) / (1.0395863 - P)) * (c + (((c + -0.37225553) * P) * (P + c))))) * 1.126988) * P) / 0.8171877)))))) + P
61	0.000214	((((P + -0.62939286) - -0.007309799) - -0.007309799) * ((-0.49237034 * P) / ((c / (1.0395863 - P)) + (P - ((c + -0.057566248) * ((((((P - c) / (P - 0.30867785)) - (((P + c) / (1.0395863 - P)) * (c + ((((c + -0.37225553) * P) * P) * (P + c))))) * 1.126988) * P) / 0.8171877)))))) + P
63	0.000208	((P + -0.62654173) * ((-0.49237034 * P) / ((c / (1.0395863 - P)) + (P - ((c + -0.057566248) * ((((((P - c) / (P - 0.30867785)) - (((c / (1.0395863 - P)) + (P - ((-0.057566248 * (1.0219532 / ((((c - 0.6367143) - 0.84921926) * P) + c))) + c))) * (c + c))) * 1.126988) * P) / 0.8171877)))))) + P
65	0.000201	(((P + -0.62654173) - -0.007309799) * ((-0.49237034 * P) / ((c / (1.0395863 - P)) + (P - ((c + -0.057566248) * ((((((P - c) / (P - 0.30867785)) - (((c / (1.0395863 - P)) + (P - ((-0.057566248 * (1.0219532 / ((((c - 0.6367143) - 0.84921926) * P) + c))) + c))) * (c + c))) * 1.126988) * P) / 0.8171877)))))) + P

69	0.0002	(((P + -0.62654173) - -0.007309799) * ((-0.49237034 * P) / ((c / (1.0395863 - P)) + (P - ((c + -0.057566248) * ((((((P - c) / (P - 0.30867785)) - (((((c / (1.0395863 - P)) + (P - ((-0.057566248 * (1.0219532 / ((((c - 0.6367143) - 0.84921926) * P) + c))) + c))) - c) + P) * (c + c))) * 1.126988) * P) / 0.8171877)))))) + P
71	0.000199	(((P + -0.62654173) - -0.007309799) * ((-0.49237034 * P) / ((c / (1.0395863 - P)) + (P - ((c + -0.057566248) * ((((((P - c) / (P - 0.30867785)) - ((((((c / (1.0395863 - P)) + (P - ((-0.057566248 * (1.0219532 / ((((c - 0.6367143) - 0.84921926) * P) + c))) + c))) - c) + P) * 1.0334535) * (c + c))) * 1.126988) * P) / 0.8171877)))))) + P
73	0.000198	(((P + -0.62654173) - -0.007309799) * ((-0.49237034 * P) / ((c / (1.0395863 - P)) + (P - ((c + -0.057566248) * ((((((P - c) / (P - 0.30867785)) - ((((((c / (1.0395863 - P)) + (P - ((-0.057566248 * (1.0219532 / ((((c - 0.6367143) - 0.84921926) * P) + c))) + c))) - (c * 0.8042093)) + P) * 1.0334535) * (c + c))) * 1.126988) * P) / 0.8171877)))))) + P
75	0.000193	(((P + -0.62939286) - -0.007309799) * ((-0.49237034 * P) / ((c / (1.0395863 - P)) + (P - ((c + -0.057566248) * ((((((P - c) / (P - 0.30867785)) - (((((c / (1.0395863 - P)) + (c - (-0.057566248 * (((1.0395863 - P) / (-0.8075994 + (0.25540924 + P))) /

		((((0.2560399 - 0.84921926) * P) + c) - -0.027343856))))) + P) - c) * (c + c))) * 1.126988) * P) / 0.8171877)))))) + P
77	0.000192	(((P + -0.62939286) - -0.007309799) * ((-0.49237034 * P) / ((c / (1.0395863 - P)) + (P - ((c + -0.057566248) * ((((((P - c) / (P - 0.30867785)) - (((((c / (1.0395863 - P)) + (0.82218134 - (-0.057566248 * (((1.0395863 - P) / (-0.8075994 + (0.25540924 + P))) / ((((0.2560399 - 0.84921926) * P) + c) - (-0.027343856 * c)))))) + P) - c) * (c + c))) * 1.126988) * P) / 0.8171877)))))) + P
79	0.000188	(((P + -0.62939286) - -0.007309799) * ((-0.49237034 * P) / ((c / (1.0395863 - P)) + (P - ((c + -0.057566248) * ((((((P - c) / (P - 0.30867785)) - (((((c / (1.0395863 - P)) + (0.82218134 - (-0.057566248 * ((((1.0395863 - P) / (-0.8075994 + (0.25540924 + P))) - P) / ((((0.2560399 - 0.84921926) * P) + c) - (-0.027343856 * c)))))) + P) - c) * (c + c))) * 1.126988) * P) / 0.8171877)))))) + P
81	0.000187	(((P + -0.62939286) - -0.007309799) * ((-0.49237034 * P) / ((c / (1.0395863 - P)) + (P - ((c + -0.057566248) * ((((((P - c) / (P - 0.30867785)) - ((((((c / (1.0395863 - P)) + (P - (-0.057566248 * ((((1.0395863 - P) / (-0.8075994 + (0.25540924 + P))) - P) / ((((0.2560399 - 0.84921926) * P) +

		c) - (-0.027343856 * c)))))) + P) - c) + P) * (c + c))) * 1.126988) * P) / 0.8171877)))))) + P
83	0.000186	(((P + -0.62939286) - -0.007309799) * ((-0.49237034 * P) / ((c / (1.0395863 - P)) + (P - ((c + -0.057566248) * ((((((P - c) / (P - 0.30867785)) - ((((((c / (1.0395863 - P)) + (P - (-0.057566248 * ((((1.0395863 - P) / (-0.8075994 + (0.25540924 + P))) - P) / ((((0.2560399 - 0.84921926) * P) + c) - (-0.027343856 * (c * 1.0899854))))))) + P) - c) + P) * (c + c))) * 1.126988) * P) / 0.8171877)))))) + P
85	0.000183	(((P + -0.62939286) - -0.007309799) * ((-0.49237034 * P) / ((c / (1.0395863 - P)) + (P - ((c + -0.057566248) * ((((((P - c) / (P - 0.30867785)) - (((((((c / (1.0395863 - P)) + (P - (-0.057566248 * ((((1.0395863 - P) / (-0.8075994 + (0.25540924 + P))) - P) / ((((0.2560399 - 0.84921926) * P) + c) - (-0.027343856 * c)))))) + P) - c) + P) * 1.315807) * ((c * c) + c))) * 1.126988) * P) / 0.8171877)))))) + P
87	0.000183	(((P + -0.62939286) - -0.007309799) * ((-0.49237034 * P) / ((c / (1.0395863 - P)) + (P - ((c + -0.057566248) * (((((((P - c) / (P - 0.30867785)) - (((((((c / (1.0395863 - P)) + (P - (-0.057566248 * ((((1.0395863 - P) / (-0.8075994 + (0.25540924 + P))) - P) / ((((0.2560399 - 0.84921926) * P) +

		c) - (-0.027343856 * c)))))) + P) - c) + P) * 1.315807) * ((c * c) + c))) - 0.036252286) * 1.126988) * P) / 0.8171877)))))) + P
89	0.000182	(((P + -0.62939286) - -0.007309799) * ((-0.49237034 * P) / ((c / (1.0395863 - P)) + (P - ((c + -0.057566248) * (((((((P - c) / (P - 0.30867785)) - (((((((c / (1.0395863 - P)) + (P - (-0.057566248 * ((((1.0395863 - P) / (-0.8075994 + (0.25540924 + P))) - P) / (((((0.2560399 - 0.84921926) * P) + c) / 1.0509788) - (-0.027343856 * c)))))) + P) - c) + P) * 1.315807) * ((c * c) + c))) - 0.036252286) * 1.126988) * P) / 0.8171877)))))) + P

Table S6. Pl formulas at different levels of complexity using configuration (c), with $\overline{M}_w$ as input data.

Complexity	Loss	Equation
1	0.003971	P
3	0.003341	P ^ 0.868399
5	0.001124	P ^ (P - -0.43367666)
6	0.001064	(P ^ 0.54112333) ^ exp(P)
7	0.000979	(sqrt(P) ^ exp(P)) + -0.018546958
8	0.000899	(P ^ sqrt(P + 0.21529123)) * 0.96757597
**9 (Best)**	0.000779	(sqrt(P) ^ exp(P)) / (c ^ -0.012673381)
12	0.000772	(sqrt(P) ^ exp(P)) / (cube(1.0004748) ^ (3.932525 / c))
14	0.000703	(sqrt(P) / (1.021772 ^ square((P / P) - c))) ^ exp(P)

Table S7. Pc formulas at different levels of complexity using configuration (a), with $\overline{M}_n$ as input data.

Complexity	Loss	Equation
1	0.000985	0.031045
3	0.000578	P * 0.048578672
5	0.000496	(P * 0.056010712) * P
7	0.00023	0.014929981 / ((c - P) + 1.0097331)
9	0.000145	0.018765885 / ((c - 0.8760315) + (0.9259339 / P))
**11 (Best)**	0.000122	0.019185985 / (c + (((0.6386159 / P) - 0.57804155) / P))
13	0.000107	0.021813622 / (c + (0.05596133 / (P * ((P + -0.49036407) * P))))
15	9.89E-05	0.020223098 / (c + ((0.042919345 / (P * (-0.48920372 + P))) / (P * P)))
17	9.51E-05	0.029550077 / ((0.08456912 / (((P + -0.49281952) * P) * P)) + (c * (c + P)))
19	8.23E-	0.009792688 / (((1.4800116 / P) / P) + ((P + ((c * c) -

	05	(2.2541559 / P))) - 0.12773435))

Table S8. Pc formulas at different levels of complexity using configuration (b), with $\overline{M}_n$ as input data.

Complexity	Loss	Equation
1	0.000985	0.031037
3	0.000578	0.048578653 * P
5	0.000496	(0.056010712 * P) * P
7	0.00023	-0.014932575 / (P - (1.0097625 + C))
9	0.000145	-0.018775465 / ((0.8766795 - C) - (0.92667615 / P))
11	0.000122	-0.019192101 / ((((-0.6407872 / P) + 0.5802851) / P) - C)
13	0.000107	-0.019804528 / ((((-0.16012268 / (P - 0.44006982)) + 0.21726513) / P) - C)
15	9.89E-05	-0.020211618 / ((-0.04277631 / ((-0.49009612 + P) * (P * (P * P)))) - C)
17	9.51E-05	-0.029566702 / (((-0.08474076 / (P * (P + -0.4924602))) / P) - (C * (C + P)))

19	9.03E-05	-0.02875278 / (((-0.05519215 / ((P + -0.4979871) * P)) / (P - 0.2717473)) - (C * (C + P)))
21	8.05E-05	-0.021175653 / ((((-0.06463139 / (P + -0.45187828)) / P) - (C + (-0.020234924 / ((P * 1.363445) - C)))) / P)
23	7.88E-05	(-0.021175653 / (((-0.050066475 / P) / ((P - 0.46501905) * P)) - ((-0.020234924 / ((P * 1.363445) - c)) + c))) * P
25	6.54E-05	-0.029845554 / (((-0.08474076 / (P * (P + -0.4924602))) / P) - ((C * (P + C)) + (-0.00777502 / (-0.24784705 - (P - C)))))
27	5.86E-05	-0.027152574 / (((((-0.06463139 / (P + -0.4849629)) / P) / P) / P) - (((P + C) * C) + (-0.00777502 / ((-0.24784705 + C) - P))))
29	5.81E-05	-0.026288366 / (((((-0.06463139 / (P + -0.4849629)) / P) / P) / P) - (P * (((P + C) * C) + (-0.0078097633 / ((-0.24784705 + C) - P)))))
31	5.76E-05	-0.027152574 / (((((-0.06463139 / (P + -0.5006327)) / P) / P) / P) - ((((P + C) * C) + ((-0.00777502 / 0.9536713) / ((-0.24784705 + C) - P))) * P))
33	5.45E-05	-0.026093727 / ((((-0.06463139 / (P + -0.5006327)) / P) / P) - (C * ((P + C) + (-0.00777502 / (((-0.06463139 / (P + -0.5128263)) + C) - ((0.72107655 * C) * C))))))
**35 (Best)**	4.35E-05	-0.026093727 / (((((-0.06463139 / (P + -0.5006327)) / P) / P) / P) - (C * ((P + C) + (-0.00777502 / ((-0.06463139 / (P + -0.5128263)) - (((0.72107655 * C) * C) - C))))))

37	4.04E-05	-0.027152574 / ((((-0.06463139 / ((P + -0.5006327) * P)) / P) / P) - ((C * ((P + C) + (-0.00777502 / (((-0.06463139 / (P + -0.5128263)) + C) - ((0.72107655 * C) * C))))) / 0.9186319))
39	3.86E-05	-0.027152574 / ((((-0.06463139 / ((P + -0.5006327) * P)) / P) / P) - (((C * P) * ((P + C) + (-0.00777502 / (((-0.06463139 / (P + -0.5128263)) + C) - ((0.72107655 * C) * C))))) / 0.9186319))
41	3.66E-05	-0.027152574 / (((((-0.06463139 / (P + -0.49009612)) / P) / P) / P) - (((C * ((P + C) + ((-0.00777502 / ((((-0.06463139 / (P + -0.5128263)) + C) / C) - (0.72107655 * C))) / C))) * P) / 0.8912127))
43	3.62E-05	-0.027152574 / (((((-0.06463139 / (P + -0.49009612)) / P) / P) / P) - (((C * ((P + (C + 0.02701663)) + ((-0.00777502 / ((((-0.06463139 / (P + -0.5128263)) + C) / C) - (0.72107655 * C))) / C))) * P) / 0.8912127))
45	3.56E-05	-0.027152574 / (((((-0.06463139 / (P + -0.5006327)) / P) / (P / 0.9577558)) / P) - (((C * ((P + C) + ((-0.00777502 / ((((-0.06463139 / (P + -0.5128263)) + C) / C) - (0.72107655 * C))) / C))) * (P * 1.0400256)) / 0.8912127))
49	3.40E-05	-0.027152574 / (((((-0.06463139 / (P + -0.5006327)) / P) / P) / P) - (C * (((((P + C) * P) + ((((-0.00777502 * 1.2027427) / (((((-0.06463139 / (P + -0.5128263)) + C) / C) / C) - 0.72107655)) / C) / C)) + (P * C)) * P)))

59	3.37E-05	-0.027152574 / (((((-0.06463139 / (P + -0.5128263)) / P) / P) / P) - (((C * P) * P) * (((P * P) + C) + (((((-0.00777502 / P) / C) / (C * P)) / (((((((-0.06463139 / (P + -0.5128263)) + C) / C) / C) / P) - (0.72107655 / P)) * 0.9358592)) + (C * P)))))
61	3.33E-05	-0.027152574 / (((((-0.06463139 / (P + (-0.5128263 - (-0.027152574 * C)))) / P) / P) / P) - (((C * P) * P) * (((P * P) + C) + (((((-0.00777502 / P) / C) / (C * P)) / ((((((-0.06463139 / (P + -0.5128263)) + C) / C) / C) / P) - (0.72107655 / P))) + (C * P)))))
63	3.32E-05	-0.027152574 / (((((-0.06463139 / (P + (-0.5128263 - ((-0.02323652 / P) * C)))) / P) / P) / P) - (((C * P) * P) * (((P * P) + C) + (((((-0.00777502 / P) / C) / (C * P)) / ((((((-0.06463139 / (P + -0.5128263)) + C) / C) / C) / P) - (0.72107655 / P))) + (C * P)))))
65	3.31E-05	-0.027152574 / (((((-0.06463139 / (P + (-0.5128263 - ((-0.02323652 / (P * P)) * C)))) / P) / P) / P) - (((C * P) * P) * (((P * P) + C) + (((((-0.00777502 / P) / C) / (C * P)) / ((((((-0.06463139 / (P + -0.5128263)) + C) / C) / C) / P) - (0.72107655 / P))) + (C * P)))))
67	3.29E-05	-0.027152574 / (((((-0.06463139 / (P + (-0.5128263 - ((-0.02323652 / ((P * 1.291621) * P)) * C)))) / P) / P) / P) - (((C * P) * P) * (((P * P) + C) + (((((-0.00777502 / P) / C) / (C * P)) /

		((((((-0.06463139 / (P + -0.5128263)) + C) / C) / C) / P) - (0.72107655 / P))) + (C * P)))))
69	3.28E-05	-0.027152574 / (((((-0.06463139 / (P + (-0.5128263 - ((-0.02323652 / (((P + C) * P) * P)) * C)))) / P) / P) / P) - (((C * P) * P) * (((P * P) + C) + (((((-0.00777502 / P) / C) / (C * P)) / ((((((-0.06463139 / (P + -0.5128263)) + C) / C) / C) / P) - (0.72107655 / P))) + (C * P)))))
71	3.24E-05	-0.027152574 / (((((-0.06463139 / (P + (-0.5128263 - ((-0.02323652 / ((P - (0.093833715 / (C / 0.3959071))) / C)) * C)))) / P) / P) / P) - (((C * P) * P) * (((P * P) + C) + (((((-0.00777502 / P) / C) / (C * P)) / ((((((-0.06463139 / (P + -0.5128263)) + C) / C) / C) / P) - (0.72107655 / P))) + (C * P)))))
73	2.90E-05	-0.027152574 / (((((-0.06463139 / (P + (-0.5128263 - ((-0.02323652 / ((P - (0.093833715 / ((C - 0.22758116) / 0.3959071))) / C)) * C)))) / P) / P) / P) - (((C * P) * P) * (((P * P) + C) + (((((-0.00777502 / P) / C) / (C * P)) / ((((((-0.06463139 / (P + -0.5128263)) + C) / C) / C) / P) - (0.72107655 / P))) + (C * P)))))

Table S9. Pc formulas at different levels of complexity using configuration (c), with $\overline{M}_n$ as input data.

Complexity	Loss	Equation
2	0.000986	square(0.17788273)
3	0.000578	0.04858097 * P
4	0.000483	exp(-2.7888622 / P)
6	0.000177	0.13873295 ^ (exp(c) / P)
**7 (Best)**	0.00014	square(0.35391712 ^ (exp(c) / P))
8	0.000129	exp((-2.0844264 / P) - (c / 0.39730966))
10	0.000126	exp((-2.1328583 / P) - (c * exp(square(P))))
11	0.000116	(0.088067636 ^ ((exp(square(P)) ^ c) / P)) / 0.6793251
12	9.80E-05	cube(cube(P) / (1.0121676 + square(square(square(P)) + c)))
13	9.49E-	cube(cube(P) / (cube(1.0121676) +

	05	square(square(square(P)) + c)))
15	9.49E-05	cube(cube(P) / (square(cube(sqrt(1.0121676))) + square(square(square(P)) + c)))

Table S10. Pc formulas at different levels of complexity using configuration (a), with $\overline{M}_w$ as input data.

Complexity	Loss	Equation
1	0.000618	0.021913
3	0.000455	P * 0.03327619
5	0.000265	(0.005431235 * P) / c
7	0.000166	0.006957374 * ((P * P) / c)
9	0.000111	(0.01150832 / ((0.053401373 + c) / P)) * P
**11 (Best)**	9.91E-05	(P / ((0.03412733 + (c * c)) / 0.0036929783)) * P
13	9.09E-05	(0.0033950962 / ((c * c) - (-0.021134831 / (P - 0.2754279)))) * P
15	8.85E-05	P * (0.0032535167 / ((P * (c * c)) - (-0.019456454 / (P - 0.3060104))))
17	7.92E-05	P * (-0.0036375655 / ((-0.04289584 / ((P + (P - 0.5427355)) - c)) - (c * c)))

19	7.38E-05	(0.0031724558 / ((c * (c - (0.013547415 / (P - 0.6396613)))) - (-0.023456726 / (P - 0.24954972)))) * P

Table S11. Pc formulas at different levels of complexity using configuration (b), with $\overline{M}_w$ as input data.

Complexity	Loss	Equation
1	0.000618	0.021913
3	0.000455	0.033276185 * P
5	0.000265	(P / c) * 0.0054312134
7	0.000166	(P / c) * (0.0069571435 * P)
9	0.000111	-0.011508805 * ((P / (-0.053411644 - C)) * P)
11	9.91E-05	(0.0036922956 * (P / (0.034129385 + (c * c)))) * P
13	9.09E-05	-0.0034066504 * (P / ((-0.021342317 / (P - 0.27319357)) - (C * C)))
15	8.56E-05	(P + (1.0999953 / ((((-0.086817324 / P) / P) / P) - c))) * -0.016224207
17	7.10E-05	-0.0031777902 * (((P / (-0.030410703 - (C * C))) - (0.29822806 / (P - 0.665414))) * P)

19	6.88E-05	-0.0040108743 * ((((-0.1965612 + P) / (-0.029140567 - (C * C))) - (0.221267 / (P - 0.6696762))) * P)
21	6.49E-05	-0.003374209 * (P * ((P / (-0.033157907 - (C * C))) - (0.26836205 / ((0.0069572083 / C) + (-0.7074769 + P)))))
**23 (Best)**	5.79E-05	-0.003374209 * (P * ((P / (-0.03232031 - (C * C))) - (0.28994772 / ((P + ((0.0069572083 / C) * 1.4599029)) + -0.7074769))))
25	5.50E-05	((P / ((-0.03232031 / P) - (c * c))) - ((0.23405215 / ((P - 0.036864825) - (0.66017604 - (0.0069572083 / c)))) - P)) * -0.0034743336
27	5.23E-05	((P / ((-0.02643641 / (P - 0.13234061)) - (c * c))) - ((0.23405215 / ((P - 0.036864825) - (0.66017604 - (0.0071923533 / c)))) - P)) * -0.0034743336
29	5.09E-05	((P / ((-0.03232031 / P) - (c * c))) - ((0.23405215 / ((P - (0.03745608 / P)) - (0.66017604 - (0.0069572083 / (c * 0.58710325))))) - P)) * -0.0034743336
31	5.05E-05	((P / (((-0.03232031 / P) + 0.0013164729) - (c * c))) - ((0.23405215 / ((P - (0.03745608 / P)) - (0.66017604 - (0.0069572083 / (c * 0.58710325))))) - P)) * -0.0034743336
33	4.95E-05	((P / ((-0.03232031 / P) - (c * c))) - (((0.23405215 * 0.89718485) / ((P - (0.0403988 * ((c - -0.3614793) / P))) -

		(0.66017604 - (0.0069572083 / c)))) - 0.83887076)) * - 0.0034743336
35	4.91E-05	((P / ((-0.03232031 / P) - (c * c))) - (((0.23405215 * 0.89718485) / ((P - (0.0403988 * ((c - -0.3614793) / P))) - (0.66017604 - (0.0069572083 / c)))) - (c + 0.43484572))) * - 0.0034743336
37	4.84E-05	((P / ((-0.03232031 / P) - (c * c))) - ((((0.23405215 * 0.89718485) / ((P - (0.0403988 * ((c - -0.3614793) / P))) - (0.66017604 - (0.0069572083 / c)))) - 0.79383343) - (P * c))) * -0.0034743336
39	4.80E-05	((P / ((-0.03232031 / P) - (c * c))) - ((((0.23405215 * 0.89718485) / ((P - (0.0403988 * ((c - -0.3614793) / P))) - (0.66017604 - (0.0069572083 / c)))) - 0.79383343) - ((P * c) * c))) * -0.0034743336
41	4.77E-05	((P / ((-0.03232031 / P) - (c * c))) - ((((0.23405215 * 0.89718485) / ((P - (0.0403988 * ((c - -0.3614793) / P))) - (0.66017604 - ((0.0069572083 * 1.0644417) / c)))) - 0.79383343) - ((P * c) * c))) * -0.0034743336
43	4.74E-05	((P / ((-0.03232031 / P) - (c * c))) - ((((0.23405215 * 0.89718485) / ((P - (0.0403988 * ((c - -0.36204985) / P))) - (0.66017604 - (0.0069572083 / (c / 1.1042411))))) - ((P + c) * c)) + (c - 0.7067418))) * -0.0034743336

45	4.74E-05	((P / ((-0.03232031 / P) - (c * c))) - ((((0.23405215 * 0.89718485) / ((P - (0.0403988 * (((c - 0.5370279) - -0.90491307) / P))) - (0.66017604 - (0.0069572083 / (c / 1.1042411))))) - ((P + c) * c)) + (c - 0.7067418))) * -0.0034743336
47	4.72E-05	((P / ((-0.03232031 / P) - (c * c))) - (((((0.23405215 * 0.89718485) / ((P - ((0.036864825 * 1.0975132) * ((c - -0.36204985) / P))) - (0.66017604 - (0.0069572083 / (c / 1.1042411))))) - 0.79383343) - ((P + P) * (c * c))) + c)) * -0.0034743336
49	4.70E-05	((P / ((-0.03232031 / P) - (c * c))) - (((((0.23405215 * 0.89718485) / ((P - ((0.036864825 * 1.0975132) * ((c - -0.36204985) / P))) - (0.66017604 - (0.0069572083 / (c / 1.1042411))))) - 0.79383343) - ((P + P) * ((c * c) / 0.84638566))) + c)) * -0.0034743336
51	4.70E-05	((P / ((-0.03232031 / P) - (c * c))) - (((((0.23405215 * 0.89718485) / ((P - ((0.036864825 * 1.0975132) * ((c - -0.36204985) / P))) - (0.66017604 - (0.0069572083 / (c / 1.1042411))))) - 0.79383343) - (((P * P) + P) * ((c * c) / 0.84638566))) + c)) * -0.0034743336
53	4.69E-05	((P / ((-0.03232031 / P) - (c * c))) - (((((0.23405215 * 0.89718485) / ((P - ((0.036864825 * 1.0975132) * ((c - (-

		0.36204985 / 0.9298905)) / P))) - (0.66017604 - (0.0069572083 / (c / 1.1170046))))) - 0.79383343) - ((P + ((c * c) * (P - -0.34537))) * c)) + c)) * -0.0034743336
55	4.68E-05	((P / ((-0.03232031 / P) - (c * c))) - (((((0.23405215 * 0.89718485) / ((P - ((0.036864825 * 1.0975132) * ((c - (-0.36204985 / 0.9298905)) / P))) - (0.66017604 - (0.0069572083 / (c / 1.1170046))))) - 0.79383343) - ((P + ((c * c) * (P - (-0.4537817 * P)))) * c)) + c)) * -0.0034743336
57	4.68E-05	(((P / ((-0.03232031 / P) - (c * c))) - (((((0.23405215 * 0.89718485) / ((P - ((0.036864825 * 1.0975132) * ((c - (-0.36204985 / 0.9298905)) / P))) - (0.66017604 - (0.0069572083 / (c / 1.1170046))))) - 0.79383343) - ((P + ((c * c) * (P - (-0.4537817 * P)))) * c)) + c)) * -0.0034743336) * 1.0126805
59	4.66E-05	((P / ((-0.03232031 / P) - (c * c))) - (((((0.23405215 * (0.89718485 + ((-0.00783713 / c) / -0.4994137))) / ((P - ((0.036864825 * 1.0975132) * ((c - (-0.36204985 / 0.9298905)) / P))) - (0.66017604 - ((0.0069572083 / (c / 1.1170046)) / 0.9900349)))) - 0.79383343) - ((P + P) * (c / 1.0279754))) + (c * 0.7009211))) * -0.0034743336
61	4.61E-05	((P / ((-0.03232031 / P) - (c * c))) - (((((0.23405215 * (0.89718485 + ((-0.00783713 / c) / -0.4994137))) / ((P -

		((0.036864825 * 1.0975132) * ((c - (-0.36204985 / 0.9298905)) / P))) - (0.66017604 - ((0.0069572083 / (c / 1.1170046)) / 0.9900349)))) - 0.79383343) - ((P + (c * P)) * (c / 1.0279754))) + (c * 0.7009211))) * -0.0034743336
65	4.60E-05	((P / ((-0.03232031 / P) - (c * c))) - (((((0.23405215 * (0.89718485 + ((-0.00783713 / c) / -0.4994137))) / ((P - ((0.036864825 * 1.0975132) * ((c - (-0.36204985 / 0.9298905)) / P))) - (0.66017604 - (0.0069572083 / (c / 1.1170046))))) - 0.79383343) - ((P + (((c * c) * (P - -0.4537817)) * P)) * (c / 1.0279754))) + (c * 0.7009211))) * -0.0034743336
67	4.59E-05	((P / ((-0.03232031 / P) - (c * c))) - (((((0.23405215 * (0.89718485 + ((-0.00783713 / c) / -0.4994137))) / ((P - ((0.036864825 * 1.0975132) * ((c - (-0.36204985 / 0.9298905)) / P))) - (0.66017604 - ((0.0069572083 / (c / 1.1170046)) / 0.9900349)))) - 0.79383343) - ((P + (((c * c) * (P - -0.4537817)) * P)) * (c / 1.0279754))) + (c * 0.7009211))) * -0.0034743336
69	4.58E-05	((P / ((-0.03232031 / P) - (c * c))) - ((((((0.23405215 * (0.89718485 + ((-0.00783713 / c) / -0.4994137))) / ((P - ((0.036864825 * 1.0975132) * ((c - (-0.36204985 / 0.9298905)) / P))) - (0.66017604 - ((0.0069572083 / (c /

		1.1170046)) / 0.9900349)))) - 0.79383343) * 0.9776383) - ((P + (((c * c) * (P - -0.4537817)) * P)) * (c / 1.0279754))) + (c * 0.7009211))) * -0.0034743336

Table S12. Pc formulas at different levels of complexity using configuration (c), with $\overline{M}_w$ as input data.

Complexity	Loss	Equation
1	0.211813	0.481472
2	0.000618	square(0.14803585)
3	0.000455	0.0332762 * P
4	0.000449	0.03745608 * square(P)
5	0.000265	0.005431349 * (P / c)
6	0.000148	(0.007993019 * cube(P)) / c
7	0.0001	0.03523008 ^ (c + (0.6712284 / P))

	27	
**8 (Best)**	0.0001 12	0.03523008 ^ (0.6712284 + (c ^ square(P)))
9	9.89E-05	0.03523008 ^ (0.6712284 + ((c ^ P) / P))
13	8.97E-05	0.07787168 ^ square((c * (P ^ P)) + (0.88093054 / sqrt(P)))
16	8.75E-05	((P ^ (0.6661745 / cube(P * P))) ^ c) * log(square(c) ^ -0.0131796785)
17	8.50E-05	cube((P - (c * (cube(sqrt(cube(cube(P)))) + sqrt(0.41552627)))) + 0.16704905) * 0.076808445
18	7.98E-05	cube(0.16704905 + (P - (c * (square(square(cube(P))) + cube(0.73102105 ^ c))))) * 0.076808445
20	7.65E-05	0.075535014 * cube((P - (c * (square(cube(square(P))) + (sqrt(0.20202921 ^ c) ^ c)))) + 0.2129468)
